# Preparation of mixed trialkyl alkylcarbonate derivatives of etidronic acid via an unusual route

**DOI:** 10.3762/bjoc.8.228

**Published:** 2012-11-20

**Authors:** Petri A Turhanen, Janne Weisell, Jouko J Vepsäläinen

**Affiliations:** 1 University of Eastern Finland, School of Pharmacy, Biocenter Kuopio, P.O. Box 1627, FIN-70211, Kuopio, Finland

**Keywords:** alkyl carbonate, bisphosphonate, etidronate, ester, synthesis

## Abstract

A method to prepare four (**3a**–**d**) trialkyl alkylcarbonate esters of etidronate from *P*,*P*'-dimethyl etidronate and alkyl chloroformate was developed by utilizing unexpected demethylation and decarboxylation reactions. The reaction with the sterically more hindered isobutyl chloroformate at a lower temperature (90 °C) produced the *P*,*P*'-diester (**2**) as a stable intermediate product. A possible reaction mechanism is discussed to explain these methyl substitutions. These unusual reactions also clarify why it is difficult to prepare alkylcarbonate prodrugs from bisphosphonates. The compounds prepared were analysed by spectroscopic techniques.

## Introduction

**Figure 1:**
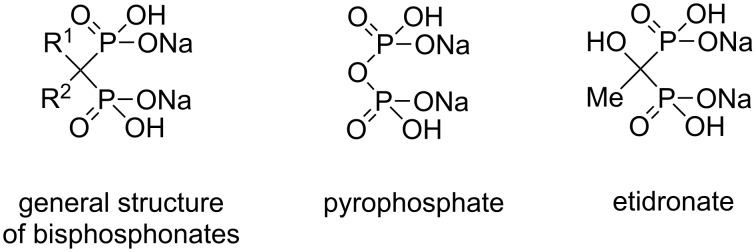
Structure of etidronate, pyrophosphate and general structure of bisphosphonates.

Bisphosphonates (BPs), which are molecules that possess the P–C–P backbone, are analogues of the naturally occurring compound pyrophosphate (Figure 1). These drugs have been used for many purposes since they were discovered ca. fifty years ago [1–3]. Initially, BPs were used as water softeners by inhibiting the crystallization of calcium salts, but today the basis for their main use is their high affinity for the bone mineral hydroxyapatite. Since they are effective inhibitors of bone resorption, BPs are used for the treatment of various bone diseases and disorders of calcium metabolism, e.g., osteoporosis [2,4]. BPs are also used as bone imaging agents when linked to a gamma-emitting technetium isotope; as bone-targeting promoieties, e.g., for anti-inflammatory drugs [5]; as solvent extraction reagents for actinide ions [6]; and as a new class of herbicides [7]. Recently, BPs have been considered as growth inhibitors for parasitic diseases such as malaria [8] and have found applications in crystal-engineering studies [9]. BP prodrugs have attracted recent interest due to their ability to inhibit isoprenoid biosynthesis [10].

Etidronate, (1-hydroxyethylidene)-1,1-bisphosphonic acid (HEBPA) disodium salt, was first synthesized over 100 years ago and is one of the most extensively investigated BP molecules (Figure 1) [1–2,4]. Our group has prepared several different HEBPA derivatives [11–17], and some of the derivatives have been considered to act as biodegradable prodrugs of etidronate [11]. Previously, we also synthesized the first phosphorous end-modified alkyl carbonate derivative of HEBPA by a surprising route [18]. According to a SciFinder search, this is the only reported phosphorous end-modified BP carbonate derivative in the scientific literature [19], while phosphorous (approximately 600 compounds) and phosphonic acid carbonates (approximately 20 compounds) are well known molecules.

Since BPs are very hydrophilic, their bioavailabilities are very poor [20]. It would be a clear advantage if one could prepare more lipophilic and biodegradable derivatives of BPs. One rather straightforward way to improve the lipophilicity of these compounds is to prepare alkyl carbonate derivatives of BPs. Alkyl carbonate derivatives of phenol and naphthols have been reported to undergo chemical and enzymatic hydrolysis [21–22] and so they have been considered as prodrugs, e.g., naltrexone [23–24], which is used for the treatment of alcohol dependence [25] and opioid addiction [26]. This was the starting point for the research project reported here.

## Results and Discussion

**Scheme 1:**
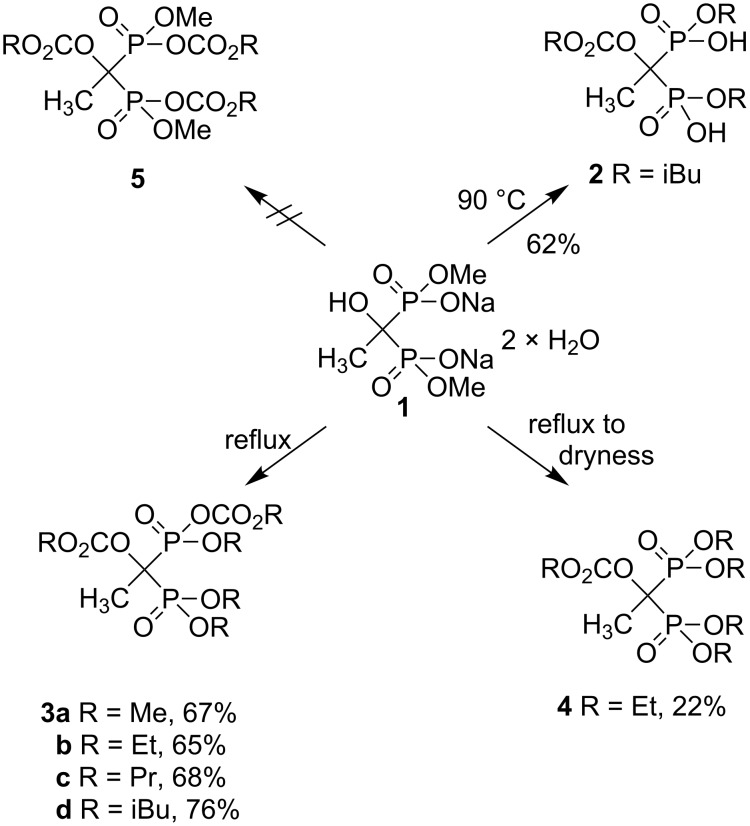
Preparation of mixed alkyl carbonate ester derivatives of HEBPA **3a–d** and diester derivative **2**. Conditions: in excess ClC(O)OR, with 3 equiv NaHCO_3_ or 4 equiv Na_2_CO_3_ (for compound **4**), overnight.

Our first goal was to prepare a mixed ethyl carbonate methyl ester derivative **5** (Scheme 1, R = Et), but unexpectedly according to the ^31^P NMR spectrum, a structure corresponding to **3** was obtained. This extraordinary synthesis route to structures such as **3** starting from *P*,*P*'-dimethyl ester of etidronate **1** led us to examine this phenomenon in a more systematic manner.

Etidronate *P*,*P*'-dimethyl ester disodium salt (**1**), which was prepared by a known method [13], was suspended in an excess of ethyl chloroformate, and subsequently 3 equiv of NaHCO_3_ were added and the reaction mixture was heated under reflux overnight. After isolation, a colorless syrup was obtained, which was surprisingly identified as compound **3b** in a series of spectroscopic experiments. Since this was such an unexpected structure, we confirmed the structure of **3b** through the following procedure: Etidronate triethyl ester mono potassium salt [13] was heated under reflux with 1 equiv of NaHCO_3_ and an excess of ethyl chloroformate in CH_3_CN for 41 h. After workup (same as when prepared by using **1** as starting material, see Experimental section), a colorless syrup was obtained and as expected the structure of the compound was verified to be **3b**, identical to the result when the synthesis was started from compound **1** (^1^H and ^31^P NMR spectra were identical).

In this extraordinary synthesis, two methyl groups and two sodium salts from the starting material **1** were changed into three ethyl and one ethoxycarbonyl groups, and also the tertiary hydroxy group was derivatized with ethoxycarbonyl. The same phenomenon was observed also when either methyl or isobutyl chloroformate was used in the reaction under reflux; invariably the three corresponding esters and one alkoxycarbonyl group were observed in the phosphorous ends with a tertiary hydroxy derived with an alkoxycarbonyl group.

**Scheme 2:**
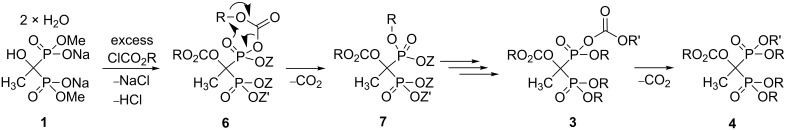
Possible reaction mechanism for conversion of **1** to **3** and **4** via key intermediates.

Another unexpected reaction was observed when compound **1** was treated with isobutyl chloroformate at 90 °C (see Scheme 2). After workup procedure from the ^31^P NMR spectrum we detected only one symmetric product, which was confirmed to be compound **2** according to a subsequent mass-spectrometric analysis.

The reaction from **1** to **3b** was also tested under milder reaction conditions, e.g., 2 equiv of ethyl chloroformate in CH_3_CN or an excess of ethyl chloroformate at room temperature, and with variable NaHCO_3_ amounts. These tests did not lead to the formation of **3b** or **4** in any reasonable yields or purities. The tetramethyl ester of etidronate was also tested as a starting material with ethyl chloroformate at 55 °C and under reflux, with no success. When the excess of propyl chloroformate was tested under reflux, product **3c** was observed, but unfortunately the purity of the product was ca. 85%. Furthermore, purification on an oven-dry (120 °C) silica column with dry eluent (dried with MgSO_4_) was not successful due to selective hydrolysis of the carbonate moiety from the phosphorus end. However, according to the ^31^P NMR spectrum, compound **3b** was stable for at least 3 h in methanol solution containing 2 equiv 1 M HCl, indicating that some kind of catalysis is needed to hydrolyze the carbonate moiety from the phosphorus end.

One possible reaction mechanism to account for this unusual conversion is shown in Scheme 2. Based on general chemical knowledge, the first intermediate in this reaction cascade must be compound **6** (Z = Me, Z’ = Na, H or CO_2_R), since in general OH and ONa groups react readily with chloroformates, forming the corresponding carbonates. However, the formed carbonate structure at the phosphorus end seems to be unstable since only rearranged products **3** could be isolated. Moreover, in the case of sterically more hindered isobutyl chloroformate, also the corresponding *P*,*P'*-diester **2** was isolated according to the ^13^C NMR spectrum and mass spectrometry.

Most probably this rearrangement from carbonate **6** to ester **7** proceeds by decarboxylation in conjunction with the 1,5-migration of an alkyl group from the carbonate oxygen to the phosphorus oxygen. There are only two studies describing this kind of phenomenon in the literature, with the first being reported in 1979 by Hewitt [27] and then being revisited by Afarinkia [28] 24 years later. However, the reaction mechanism proposed by Afarinkia [28] is not possible in our case. It seems more likely that the observed rearrangement is based on a six-membered transition state, in which the anchimeric assistance of the P=O group permits 1,5-alkyl migration and irreversible decarboxylation, leading to intermediate **7**. Similar pyrolytic elimination reactions were studied intensively ca. 60 years ago for carbon backbones [29]. Methyl groups from intermediate **7** (Z = Me, Z’ = H, Na or R) are possibly hydrolyzed either before rearrangement following a similar reaction route reported previously, when *P*,*P*-diesters of clodronic acid were prepared from the corresponding triesters by using mesyl chloride as the reagent [30], or more probably after rearrangement by means of a regular acid (HCl) hydrolysis. The driving force in this irreversible reaction is entropy, since gaseous CH_3_Cl is evaporated leading to product **2**. The formation of the stable intermediate **2** also explains the difficulty encountered in preparing carbonate prodrugs from bisphosphonates, since at lower temperatures the reaction does not occur, and the 1,5-alkyl migration associated with the decarboxylation occurs at elevated temperatures. The stable end product **3**, typically with yields of 65–76%, is obtained after the addition of two formate groups to intermediate **7** (Z = H, Z’ = R) followed by only one further 1,5-alkyl migration and decarboxylation. However, it is not clear why the last carbonate moiety remains leading selectively to **3** even with prolongation of the heating period. We were also able to prepare and isolate tetraester **4** (R = Et), but only at a 22% yield, when **1** was first heated under reflux in excess ethyl chloroformate with 4 equiv Na_2_CO_3_ for ca. 4 hours, followed by turning the water cooling from the reflux condenser to the minimum. With overnight stirring at ca. 100 °C, the reaction mixture was evaporated almost to dryness yielding product **4** after purification of the reaction mixture.

It can be proposed that decarboxylation of product **3** leads to tetraester derivative **4**; however, also competitive elimination reactions occur with longer carbon chains, since heating of **3c** for 2 h at 100 °C in an oil bath without any solvent produced the corresponding triester, not tetraester, according to the ^31^P NMR spectrum. We also tried to prepare a mixed tetraester derivative from **3c** by heating under reflux with absolute ethanol, but no reactions were observed to occur. All crude reaction mixtures from the synthesis of **3** from **1** contained a few per cent of the corresponding tetraester derivative as a byproduct.

**Scheme 3:**
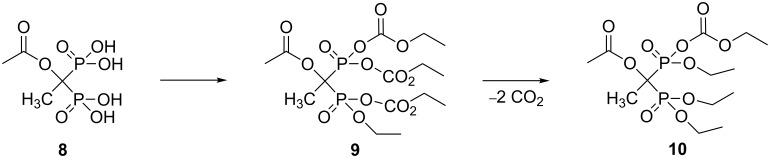
Proposed formation of mono ethylcarbonate triethyl ester derivative **10** of acetylated etidronic acid.

Previously, we reported the synthesis of compound **9** from the corresponding acetylated etidronic acid **8** (see Scheme 3) [18]. Based on the present study, we re-evaluated the spectral data from the previously reported compound **9**. The easiest method to differentiate compounds **9** and **10** from each other is by mass spectrometry, since the ^1^H, ^13^C and ^31^P NMR spectral data from these compounds are almost identical. According to re-evaluated mass spectral data, the rearranged compound **10** (C_13_H_26_O_10_P_2_ + Na, *m*/*z* = 427) and its major fragmentation at *m*/*z* = 332 were the major peaks in the positive-mode mass spectrum. Interestingly, the signal from expected compound **9** (C_15_H_26_O_14_P_2_ + Na, *m*/*z* = 515) was not seen in the positive mode; however, in the negative mode signals *m*/*z* = 332 and 515 were the major peaks, but intensities were ca. hundred times weaker compared to the positive-mode signals. Therefore, we conclude that most of the sample reported earlier [18] was the rearranged product **10** and there was a very small amount of the original product **9** left (which we wrongly interpreted as the main product, see Scheme 3).

## Conclusion

Novel mixed trialkyl alkylcarbonate ester derivatives of HEBPA (**3a**–**d**) were prepared with good yields (65–76%) via an unusual demethylation, decarboxylation and 1,5-migration route. In addition a novel *P*,*P'*-diester alkylcarbonate derivative **2** was isolated at a reasonable 62% yield and the tetraester derivative **4** was also isolated. The observed results from this study explain some of the difficulties encountered in preparing carbonate prodrugs of bisphosphonates similar to etidronate. On the other hand, the Hewitt reaction observed here enabled the preparation of more complex bisphosphonate esters from rather readily available starting materials.

## Experimental

^1^H, ^31^P and ^13^C NMR spectra were recorded with a 500 MHz spectrometer operating at 500.1, 202.5 and 125.8 MHz, respectively. The solvent residual peak was used as a standard for ^1^H and ^13^C measurements (7.26 ppm or 77.16 ppm for CDCl_3_ and 3.31 ppm or 49.00 ppm for CD_3_OD) [31], and 85% H_3_PO_4_ was used as an external standard for ^31^P measurements. The *^n^J*_HP_ couplings were calculated from proton spectra and all *J* values are given in hertz (Hz). The *^n^J*_CP_ couplings were calculated from carbon spectra with the coupling constants given in parenthesis as hertz. The purity of the products was determined from ^1^H and ^31^P NMR spectra and was ≥95% for compounds **3a**,**b**, and **2**, and ca. 85% for compounds **3c** and **3d**, which was adequate for our studies. The molar mass for compound **1** was calculated to be 278.04 though it most probably exists as a dihydrate [32]. Diastereomeric ratios for each of the compounds **3a**–**d** were calculated from the ^31^P NMR spectrum. Mass spectra were recorded on a quadrupole time-of-flight mass spectrometer by using electrospray ionization (ESI) with positive-ionization mode for compounds **3a**–**d** and **4** and negative for compound **2**. All reactions were performed in an oil bath under a nitrogen atmosphere.

**Procedure for the preparation of 3a**–**d:** Etidronate *P*,*P*'-dimethyl ester disodium salt (**1**) (100 mg, 0.36 mmol) was suspended in alkyl chloroformate (3 mL), dry NaHCO_3_ (90 mg, 3 equiv, 1.07 mmol) was added, and the mixture was heated under reflux overnight before evaporation to dryness in vacuo. The residue was suspended in diethyl ether (ca. 8 mL), the solids were removed by centrifugation, and diethyl ether was evaporated in vacuo. The products were present as colorless oils with 65–76% yields.

**Preparation of 2:** Compound **2** was prepared as above, except that the reaction temperature was 90 °C and the product obtained was a colorless amorphous solid.

**Procedure for the preparation of 4:** Etidronate *P*,*P*'-dimethyl ester disodium salt (**1**) (150 mg, 0.54 mmol) was suspended in alkyl chloroformate (3 mL), Na_2_CO_3_ (229 mg, 4 equiv, 2.16 mmol) was added, and the mixture was heated under reflux for ca. 4 h, before the water cooling was turned to a minimum in the reflux condenser. The reaction mixture was evaporated almost to dryness during the overnight stirring at ca. 100 °C. The residue was suspended to diethyl ether (ca. 8 mL), the solids were removed by centrifugation, and diethyl ether was evaporated in vacuo. The crude product was purified by silica column chromatography with EtOAc/MeOH (9:1) as eluent. Compound **4** (46 mg, 22% yield) was present as a colorless viscous oil.

**[1-Methoxycarbonyloxy-1-(methoxy-methoxycarbonyloxyphosphoryl)ethyl]phosphonic acid dimethyl ester (3a):** Pair of diastereomers (ratio ca. 60:40). Yield: 88 mg, 67%. ^1^H NMR (500.1 MHz, CDCl_3_) δ 4.04–3.99 (m, 3H), 3.92–3.84 (m, 9H), 3.80–3.78 (m, 3H), 1.979 (dd, ^3^*J*_HP_ = 16.0, ^3^*J*_HP'_ = 17.0 Hz) and 1.973 (dd, ^3^*J*_HP_ = 15.5, ^3^*J*_HP'_ = 17.0 Hz, 3H); ^13^C NMR (125.8 MHz, CDCl_3_) δ 153.36 (t, ^2^*J*_CP_ = 8.6 Hz), 153.35 (t, ^2^*J*_CP_ = 9.4 Hz), 148.25 (d, ^2^*J*_CP_ = 6.8 Hz), 148.20 (d, ^2^*J*_CP_ = 7.0 Hz), 80.0 (dd, ^1^*J*_CP_ = 155.8, ^1^*J*_CP'_ = 158.8 Hz), 79.9 (dd, ^1^*J*_CP_ = 155.6, ^1^*J*_CP'_ = 160.1 Hz), 56.36 (2C), 56.32 (d, ^2^*J*_CP_ = 6.8 Hz), 56.30 (d, ^2^*J*_CP_ = 7.5 Hz), 55.60, 55.56, 55.28 (d, ^2^*J*_CP_ = 6.7 Hz), 55.25 (d, ^2^*J*_CP_ = 6.8 Hz), 55.21 (d, ^2^*J*_CP_ = 7.2 Hz), 54.7 (d, ^2^*J*_CP_ = 7.2 Hz), 18.4 (t, ^2^*J*_CP_ = 2.5 Hz), 18.0 (t, ^2^*J*_CP_ = 2.3 Hz); ^31^P NMR (202.5 MHz, CDCl_3_) δ 17.11 (d, ^2^*J*_PP_ = 21.9 Hz), 13.38 and 17.07 (d, ^2^*J*_PP_ = 22.7 Hz), 13.39 (d); HRMS–ESI (*m*/*z*): [M + Na]^+^ calcd for C_9_H_18_O_11_P_2_Na, 387.0222; found, 387.0264.

**[1-Ethoxycarbonyloxy-1-(ethoxy-ethoxycarbonyloxyphosphoryl)ethyl]phosphonic acid diethyl ester (3b):** Pair of diastereomers (ratio ca. 50:50). Yield: 101 mg, 65%. ^1^H NMR (500.1 MHz, CDCl_3_) δ 4.50–4.43 (m, 2H), 4.36–4.19 (m, 8H), 2.02 (dd, ^3^*J*_HP_ = 16.0, ^3^*J*_HP'_ = 16.5 Hz, 3H), 1.42–1.30 (m, 15H); ^13^C NMR (125.8 MHz, CDCl_3_) δ 152.81 (t, ^2^*J*_CP_ = 9.4 Hz), 152.76 (t, ^2^*J*_CP_ = 9.4 Hz), 147.60 (d, ^2^*J*_CP_ = 6.6 Hz), 147.55 (d, ^2^*J*_CP_ = 6.6 Hz), 79.84 (dd, ^1^*J*_CP_ = 155.1, ^1^*J*_CP'_ = 158.5 Hz), 79.77 (dd, ^1^*J*_CP_ = 155.0, ^1^*J*_CP'_ = 159.7 Hz), 66.4 (d, 2C, ^2^*J*_CP_ = 7.6 Hz), 65.89, 65.88, 64.85, 64.84, 64.6 (d, ^2^*J*_CP_ = 6.8 Hz), 64.5 (d, ^2^*J*_CP_ = 6.9 Hz), 64.4 (d, ^2^*J*_CP_ = 7.2 Hz), 64.2 (d, ^2^*J*_CP_ = 7.3 Hz), 18.4 (t, ^2^*J*_CP_ = 2.2 Hz), 18.1 (t, ^2^*J*_CP_ = 2.1 Hz), 16.4 (d, 4C, ^3^*J*_CP_ = 5.9 Hz), 16.01 (d, ^3^*J*_CP_ = 6.6 Hz), 15.98 (d, ^3^*J*_CP_ = 6.6 Hz), 14.2 (2C), 14.0 (2C); ^31^P NMR (202.5 MHz, CDCl_3_) δ 14.94 (d, ^2^*J*_PP_ = 22.1 Hz), 12.06 and 14.91 (d, ^2^*J*_PP_ = 21.7 Hz), 12.11 (d); HRMS–ESI (*m*/*z*): [M + Na]^+^ calcd for C_14_H_28_O_11_P_2_Na, 457.1005; found, 457.1011.

**[1-Propoxycarbonyloxy-1-(propoxy-propoxycarbonyloxyphosphoryl)ethyl]phosphonic acid dipropyl ester (3c):** Pair of diastereomers (ratio ca. 50:50). Yield: 247 mg, 68% (starting material **1** was used (200 mg). ^1^H NMR (500.1 MHz, CDCl_3_) δ 4.34 (q, ^3^*J*_HH_ = 7.0 Hz, 2H), 4.23–4.05 (m, 8H), 2.02 (dd, ^3^*J*_HP_ = 15.5, ^3^*J*_HP'_ = 17.0 Hz, 3H), 1.79–1.65 (m, 10H), 0.99–0.92 (m, 15H); ^13^C NMR (125.8 MHz, CDCl_3_) δ 153.02 (t, ^3^*J*_CP_ = 9.1 Hz), 152.98 (t, ^3^*J*_CP_ = 8.8 Hz), 147.83 (t, ^2^*J*_CP_ = 6.9 Hz), 80.11 (dd, ^1^*J*_CP_ = 155.6, ^1^*J*_CP'_ = 158.1 Hz), 80.00 (dd, ^1^*J*_CP_ = 155.4, ^1^*J*_CP'_ = 159.7 Hz), 71.72, 71.71, 71.66, 71.64, 71.47, 70.46, 70.44, 70.10, 70.05, 70.00, 69.96, 69.92, 69.86, 69.65, 69.59, 69.57, 24.03, 23.99, 23.67, 23.62, 22.18, 22.06, 21.85, 18.55, 18.25, 10.32, 10.23, 10.19, 10.15, 10.11, 10.01, 9.99; ^31^P NMR (202.5 MHz, CDCl_3_) δ 14.89 (d, ^2^*J*_PP_ = 21.6 Hz), 12.17 and 14.86 (d, ^2^*J*_PP_ = 21.6 Hz), 12.11 (d); HRMS–ESI (*m*/*z*): [M + Na]^+^ calcd for C_19_H_38_O_11_P_2_Na, 527.1787; found, 527.1788.

**[1-Isobutoxycarbonyloxy-1-(isobutoxy-isobutoxycarbonyloxyphosphoryl)ethyl]phosphonic acid diisobutyl ester (3d):** Pair of diastereomers (ratio ca. 55:45). Yield: 157 mg, 76%. ^1^H NMR (500.1 MHz, CDCl_3_) δ 4.16 (t, ^3^*J*_HH_ = 6.8 Hz, 2H), 4.06–3.88 (m, 8H), 2.08–1.92 (m, 8H), 1.00–0.90 (m, 30H); ^13^C NMR (125.8 MHz, CDCl_3_) δ 152.88 (dd, ^3^*J*_CP_ = 10.5 and 8.2 Hz), 152.83 (dd, ^3^*J*_CP_ = 9.6 and 8.6 Hz), 147.79 (d, ^2^*J*_CP_ = 8.8 Hz), 147.73 (d, ^2^*J*_CP_ = 8.8 Hz), 80.1 (dd, ^1^*J*_CP_ = 155.9, ^1^*J*_CP'_ = 157.2 Hz), 80.0 (dd, ^1^*J*_CP_ = 155.9, ^1^*J*_CP'_ = 159.7 Hz), 75.76, 75.73, 75.72, 75.69, 75.63, 74.73, 74.71, 74.28, 74.22, 74.21, 74.15, 74.13, 74.07, 74.02, 73.97, 73.82, 73.76, 29.30, 29.26, 29.20, 29.16, 29.01, 28.96, 27.82, 27.74, 27.68, 27.59, 19.00, 18.94, 18.84, 18.83, 18.80, 18.73, 18.69, 18.68, 18.65, 18.61, 18.60, 18.57, 18.55, 18.45 (br), 18.2 (br); ^31^P NMR (202.5 MHz, CDCl_3_) δ 14.64 (d, ^2^*J*_PP_ = 21.3 Hz), 12.07 and 14.60 (d, ^2^*J*_PP_ = 21.7 Hz), 11.82 (d); HRMS–ESI (*m*/*z*): [M + Na]^+^ calcd. for C_24_H_48_O_11_P_2_Na, 597.2570; found, 597.2576.

**(1-Isobutoxycarbonyloxyethylidene)-1,1-bisphosphonic acid *P*,*P*'-diisobutylester (2):** Yield: 93 mg, 62%. ^1^H NMR (500.1 MHz, CD_3_OD) δ 3.95–3.88 (m, 6H), 1.93 (t,^3^*J*_HP_ = 15.0 Hz, 3H), 2.00–1.90 (m, 3H), 1.00–0.92 (m, 18H); ^13^C NMR (125.8 MHz, CD_3_OD) δ 154.7 (t, ^3^*J*_CP_ = 8.1 Hz), 81.5 (t, ^1^*J*_CP_ = 154.5 Hz), 75.4, 74.5 (t, ^2^*J*_CP_ = 3.3 Hz, 2C), 30.5 (t, ^3^*J*_CP_ = 2.6 Hz, 2C), 29.0, 19.20 (2C), 19.15 (4C), 19.07; ^31^P NMR (202.5 MHz, CD_3_OD) δ 14.51; HRMS–ESI (*m*/*z*): [M − H]^−^ calcd for C_15_H_31_O_9_P_2_, 417.1443; found, 417.1455.

**(1-Ethoxycarbonyloxyethylidene)-1,1-bisphosphonic acid tetraethyl ester (4):** Yield: 46 mg, 22%. ^1^H NMR (500.1 MHz, CDCl_3_) δ 4.30–4.18 (m, 8H), 4.15 (q, ^3^*J*_HH_ = 7.0 Hz, 2H), 1.92 (t, ^3^*J*_HP_ = 15.5 Hz, 3H), 1.312 (t, ^3^*J*_HH_ = 7.0 Hz, 6H), 1.307 (t, ^3^*J*_HH_ = 7.0 Hz, 6H), 1.26 (t, ^3^*J*_HH_ = 7.0 Hz, 3H); ^13^C NMR (125.8 MHz, CDCl_3_) δ 152.9 (t, ^3^*J*_CP_ = 8.9 Hz), 80.4 (t, ^1^*J*_CP_ = 154.9 Hz), 64.5, 64.2 (t, ^2^*J*_CP_ = 3.3 Hz, 2C), 63.9 (t, ^2^*J*_CP_ = 3.4 Hz, 2C) 18.5 (t, ^2^*J*_CP_ = 2.0 Hz), 16.47 (t, ^3^*J*_CP_ = 2.8 Hz, 2C), 16.46 (t, ^3^*J*_CP_ = 2.8 Hz, 2C), 14.3; ^31^P NMR (202.5 MHz, CDCl_3_) δ 16.45; HRMS–ESI (*m*/*z*): [M + Na]^+^ calcd for C_13_H_28_O_9_P_2_Na, 413.1106; found, 413.1091.

## Supporting Information

File 1 ^1^H, ^13^C and ^31^P NMR spectra for the compounds **2**, **3a–d** and **4**.
