# Supplementary material for: Preparation of mixed trialkyl alkylcarbonate derivatives of etidronic acid via an unusual route
Source: Beilstein J Org Chem. 2012 Nov 20;8:2019–24. doi: 10.3762/bjoc.8.228 (PMC3511037; doi:10.3762/bjoc.8.228)
Supplement: File 1 — 1H, 13C and 31P NMR spectra for the compounds 2, 3a–d and 4. [file Beilstein_J_Org_Chem-08-2019-s001.pdf]

Supporting Information
for
Preparation of mixed trialkyl alkylcarbonate derivatives of etidronic acid via an unusual route

Petri A. Turhanen*, Janne Weisell and Jouko J. Vepsäläinen

Address: University of Eastern Finland, School of Pharmacy, Biocenter Kuopio, P.O. Box 1627, FIN-70211, Kuopio, Finland

E-mail: Petri A. Turhanen - Petri.Turhanen@uef.fi

*Corresponding author

^{1}H, ^{13}C and ^{31}P NMR spectra for the compounds **2**, **3a–d** and **4**

Table of Contents

^{1}H, ^{31}P and ^{13}C NMR spectra of **3a** S2
^{1}H, ^{31}P and ^{13}C NMR spectra of **3b** (prepared from triethylester of etidronate) S5
^{1}H and ^{31}P NMR of **3b** (prepared as outlined in Scheme 1) S8
^{1}H, ^{31}P and ^{13}C NMR spectra of **3c** S10
^{1}H, ^{31}P and ^{13}C NMR spectra of **3d** S13
^{1}H, ^{31}P and ^{13}C NMR spectra of **2** S16
^{1}H, ^{31}P and ^{13}C NMR spectra of **4** S19

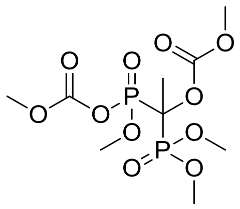

3a

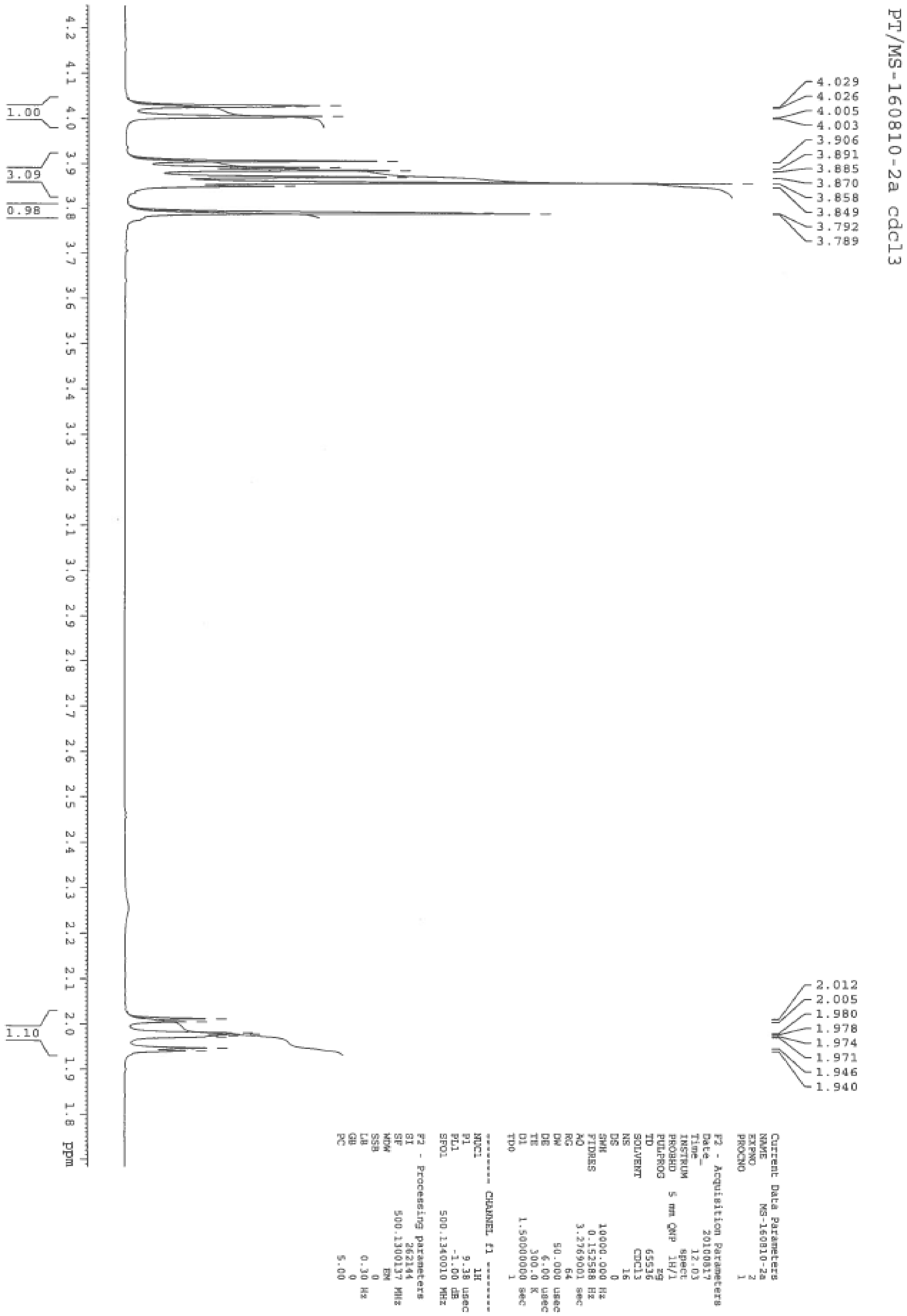
PT/MS-160810-2a cdcl3
4.029
4.026
4.005
4.003
3.906
3.891
3.885
3.870
3.858
3.849
3.792
3.789
2.012
2.005
1.980
1.978
1.974
1.971
1.946
1.940
1.00
3.09
0.98
1.10
ppm

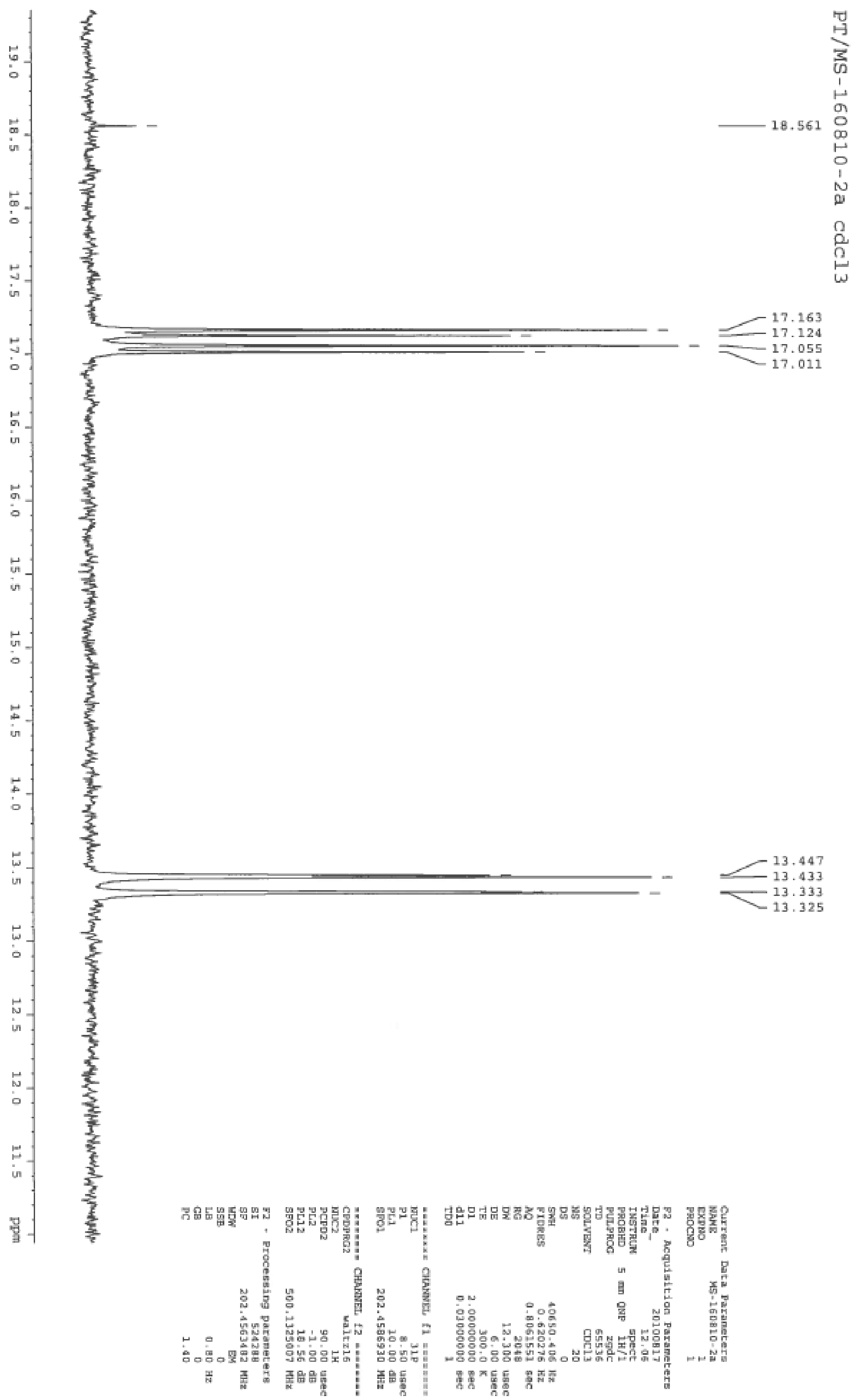
PT/MS-160810-2a cdcl3
18.561
17.163
17.124
17.055
17.011
13.447
13.433
13.333
13.325
19.0 18.5 18.0 17.5 17.0 16.5 16.0 15.5 15.0 14.5 14.0 13.5 13.0 12.5 12.0 11.5 ppm
Current Data Parameters
NAME MS-160810-2a
EXPNO 1
PROCNO 1
F2 - Acquisition Parameters
Date_ 20100817
Time 12.06
INSTRUM spect
PROBHD 5 mm QNP 1H/1
PULPROG zgdc
TD 65536
SOLVENT CDCl3
NS 20
DS 0
SWH 40650.406 Hz
FIDRES 0.620276 Hz
AQ 0.8061551 sec
RG 2048
DW 12.300 usec
DE 6.00 usec
TE 300.0 K
D1 2.00000000 sec
d11 0.03000000 sec
TD0 1
======== CHANNEL f1 ========
NUC1 31P
P1 8.50 usec
PL1 10.00 dB
SFO1 202.4586930 MHz
======== CHANNEL f2 ========
CPDPRG2 waltz16
NUC2 1H
PCPD2 90.00 usec
PL2 -1.00 dB
PL12 18.56 dB
SFO2 500.1325007 MHz
F2 - Processing parameters
SI 524288
SF 202.4563482 MHz
WDW EM
SSB 0
LB 0.80 Hz
GB 0
PC 1.40

PT/MS-160810-2a cdcl3

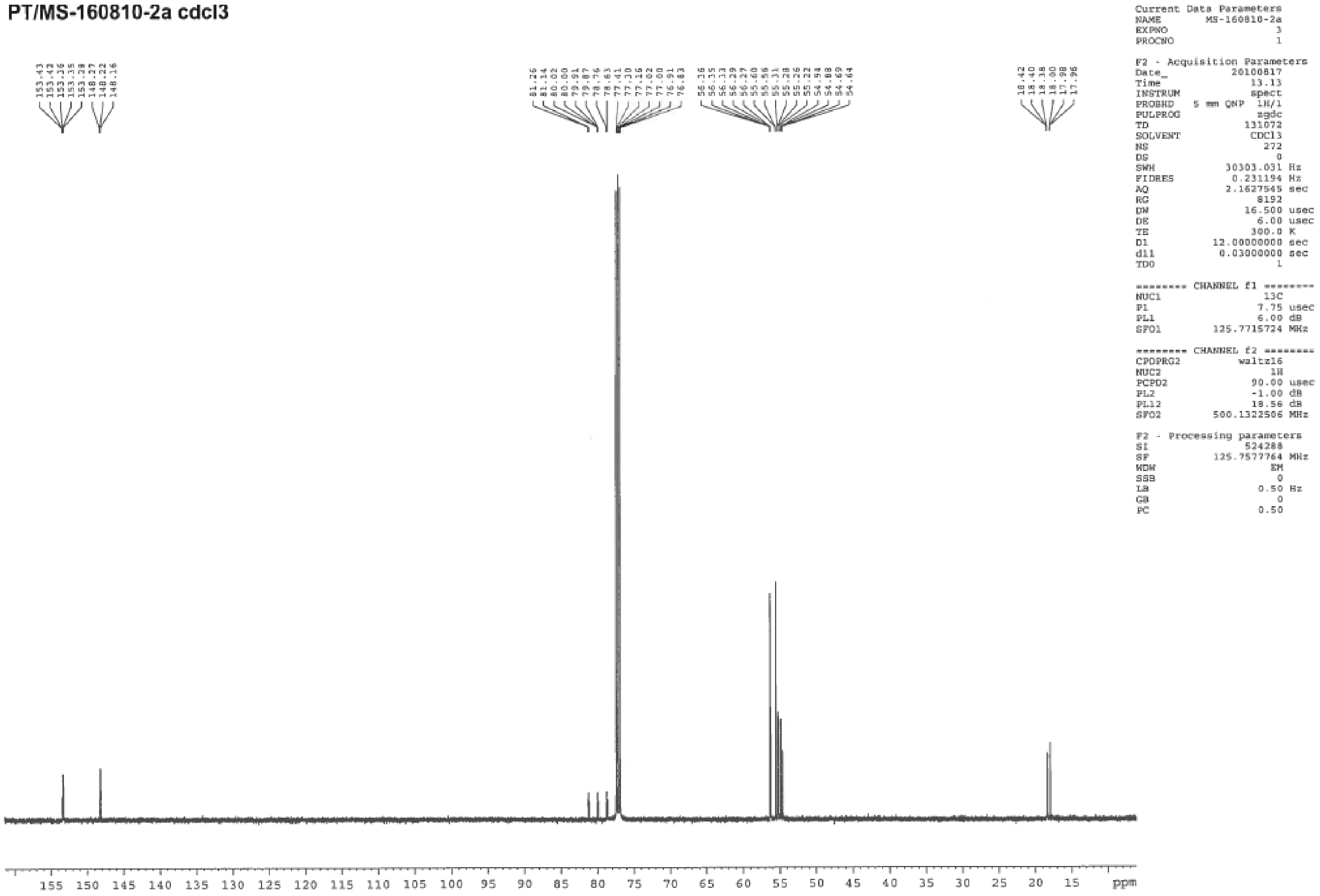

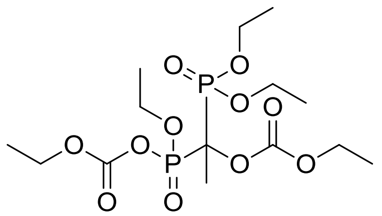

3b

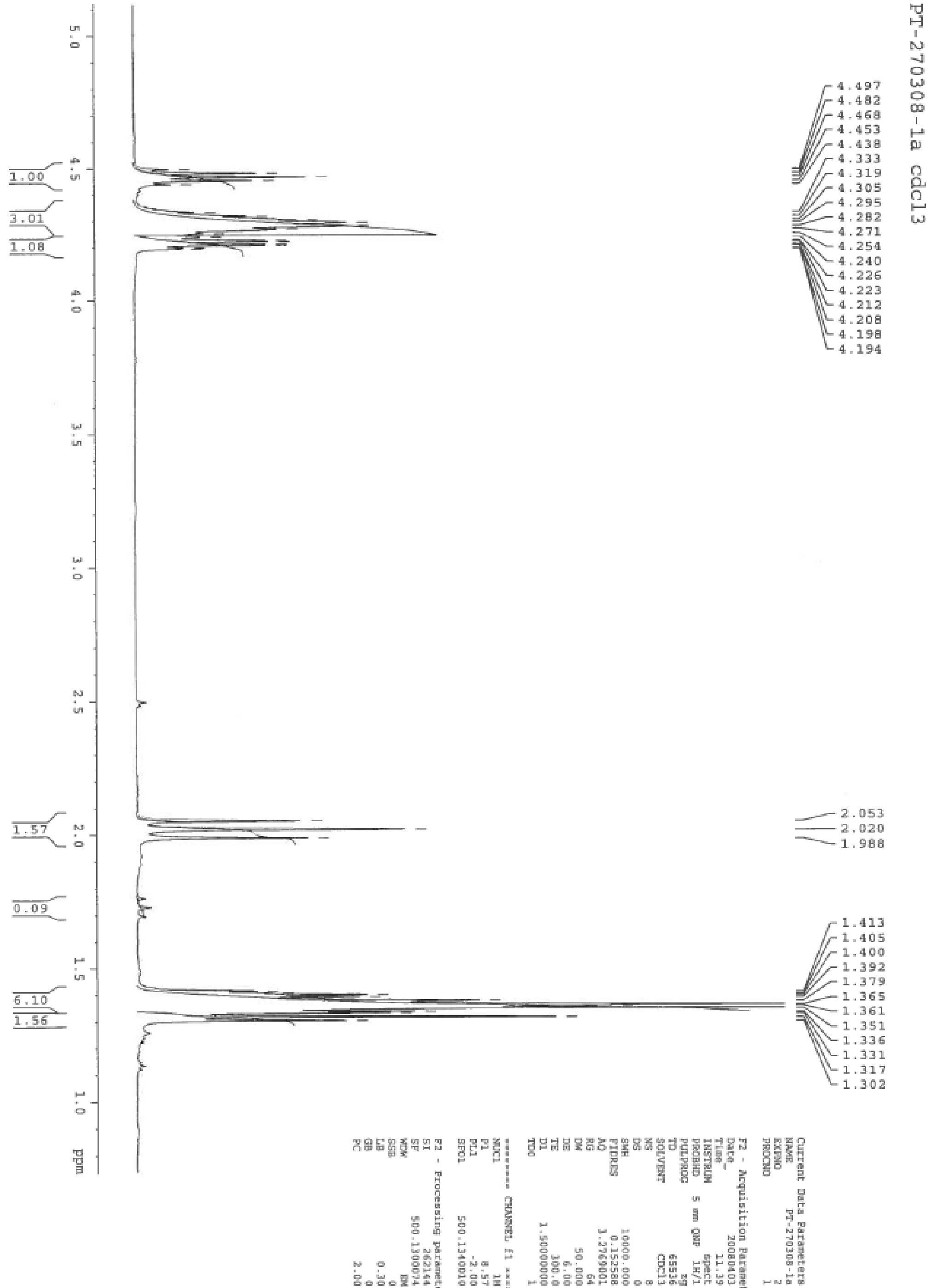
PT-270308-1a cdcl3
4.497
4.482
4.468
4.453
4.438
4.333
4.319
4.305
4.295
4.282
4.271
4.254
4.240
4.226
4.223
4.212
4.208
4.198
4.194
2.053
2.020
1.988
1.413
1.405
1.400
1.392
1.379
1.365
1.361
1.351
1.336
1.331
1.317
1.302
1.00
3.01
1.08
1.57
0.09
6.10
1.56
5.0
4.5
4.0
3.5
3.0
2.5
2.0
1.5
1.0
ppm

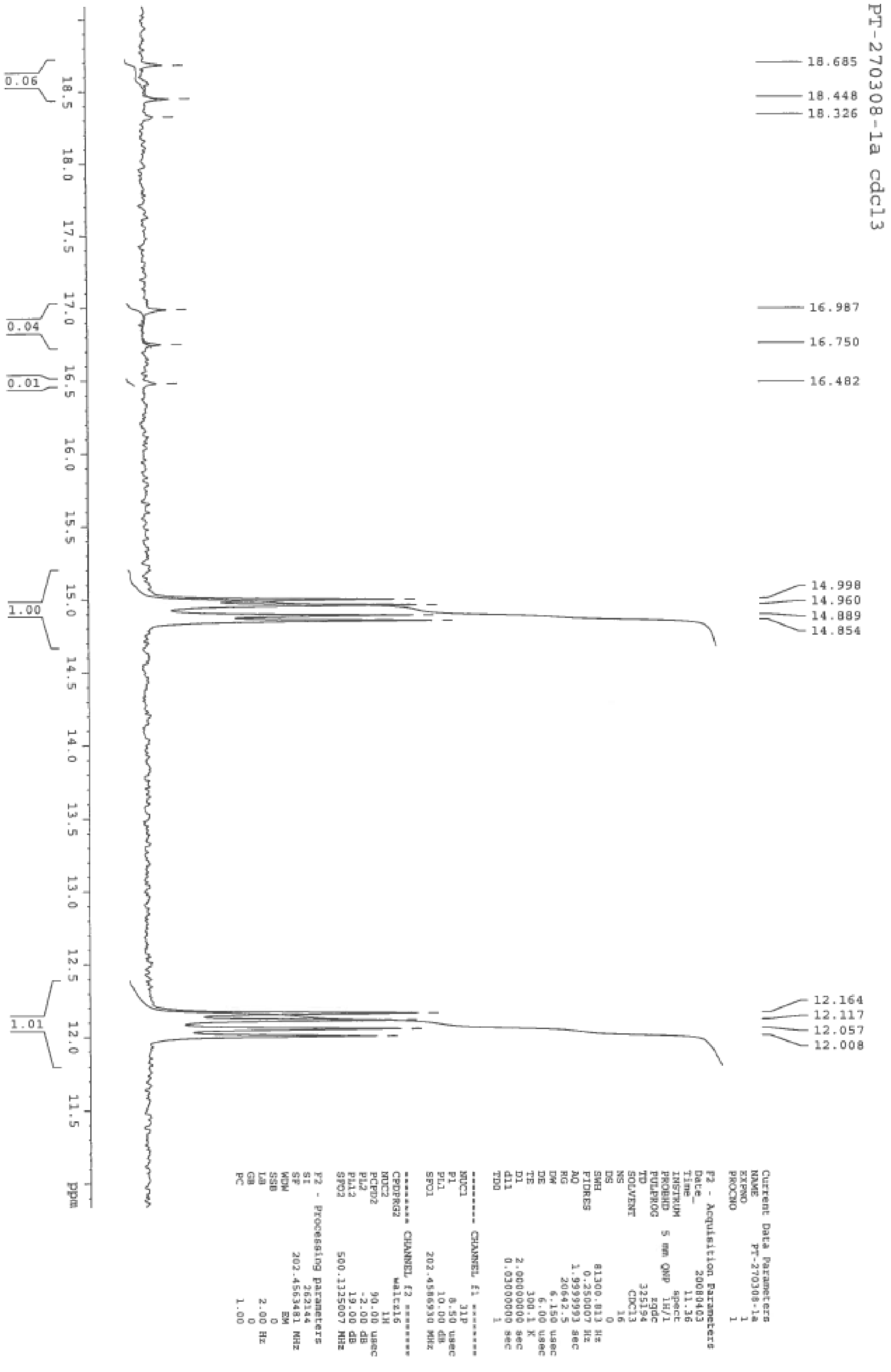
PT-270308-1a cdcl3
18.685
18.448
18.326
16.987
16.750
16.482
14.998
14.960
14.889
14.854
12.164
12.117
12.057
12.008
0.06
0.04
0.01
1.00
1.01
ppm

PT-270308-1a cdcl3

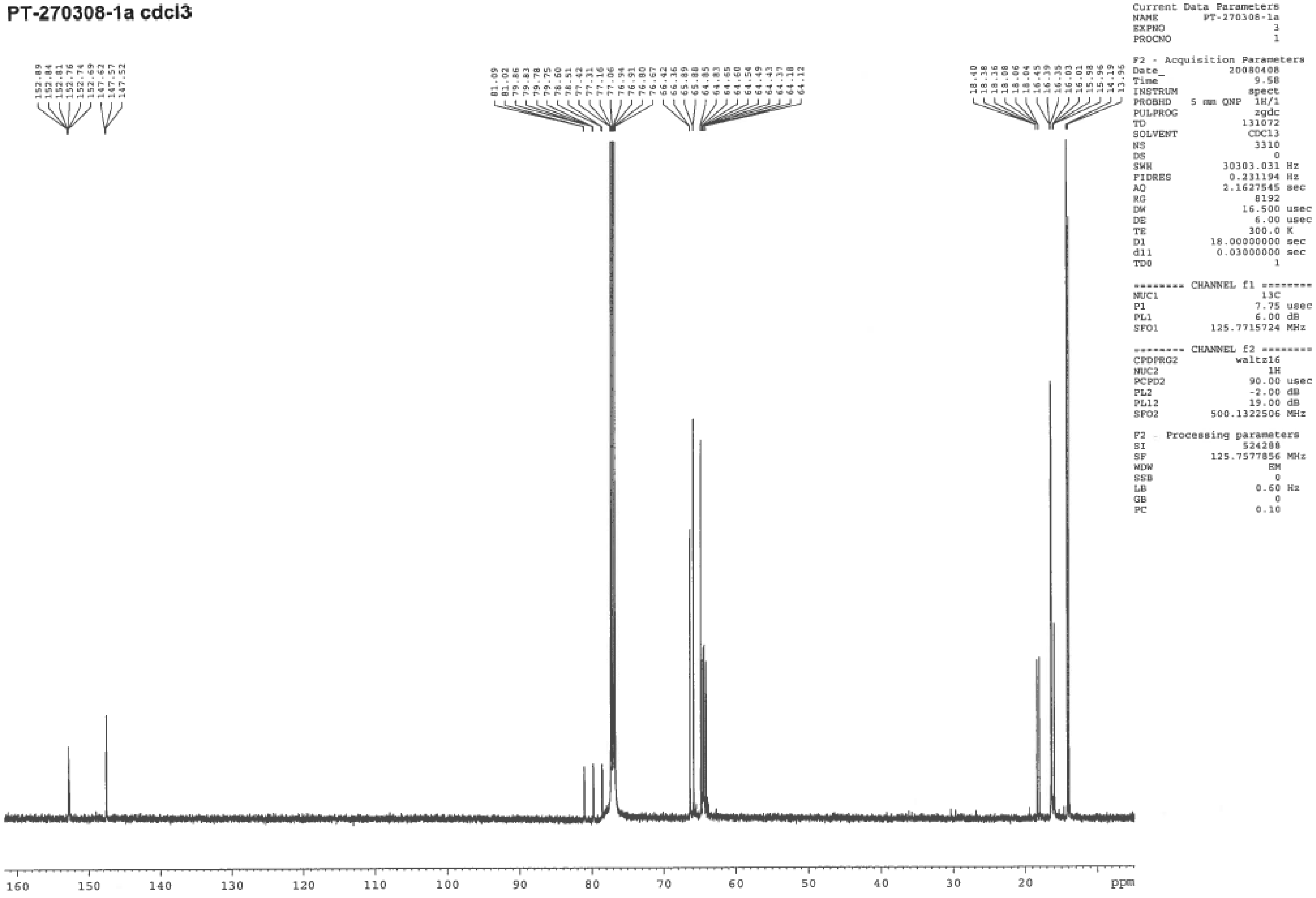

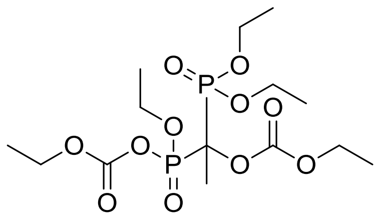

3b

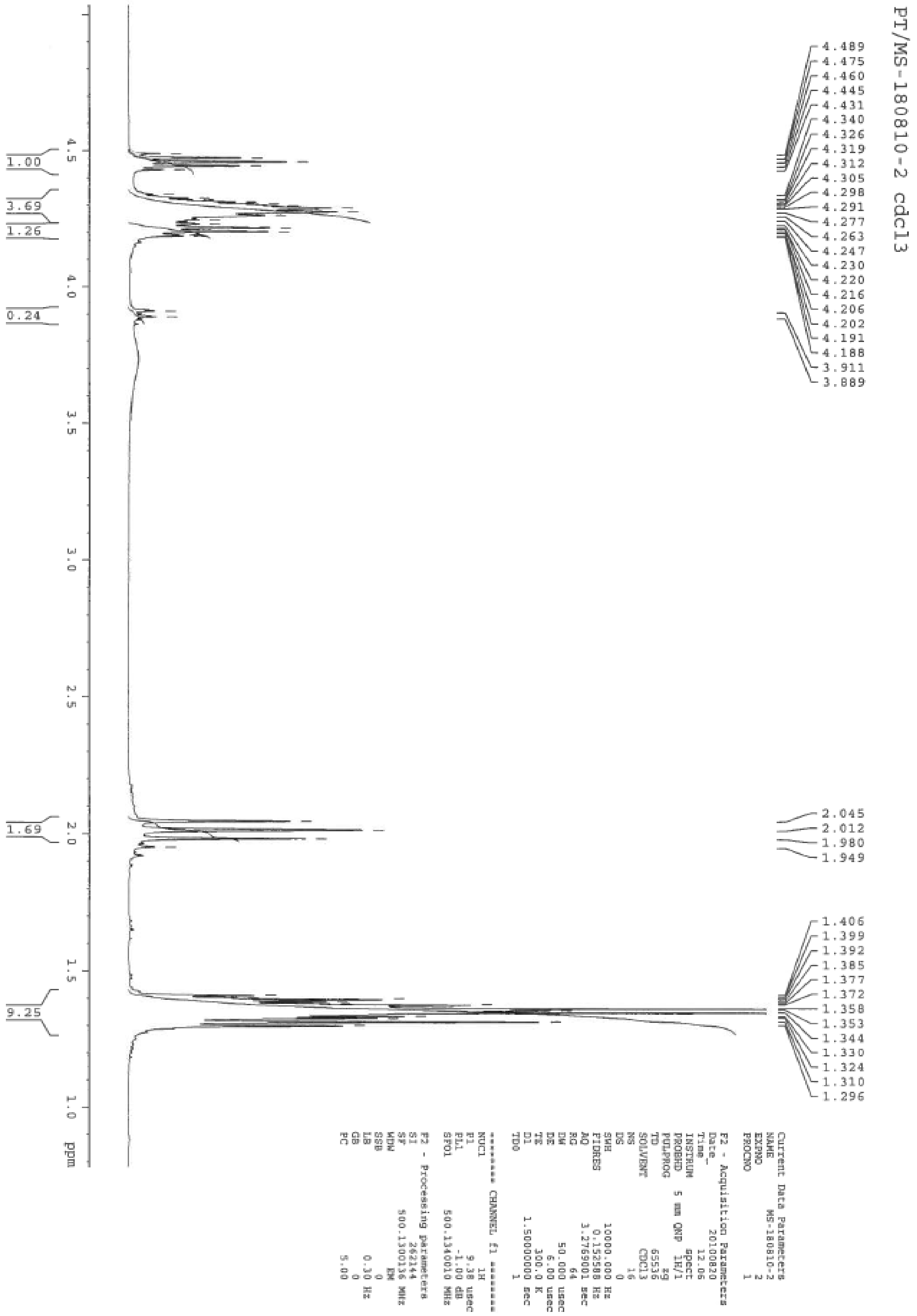
PT/MS-180810-2 cdcl3
4.489
4.475
4.460
4.445
4.431
4.340
4.326
4.319
4.312
4.305
4.298
4.291
4.277
4.263
4.247
4.230
4.220
4.216
4.206
4.202
4.191
4.188
3.911
3.889
2.045
2.012
1.980
1.949
1.406
1.399
1.392
1.385
1.377
1.372
1.358
1.353
1.344
1.330
1.324
1.310
1.296
1.00
3.69
1.26
0.24
1.69
9.25
ppm

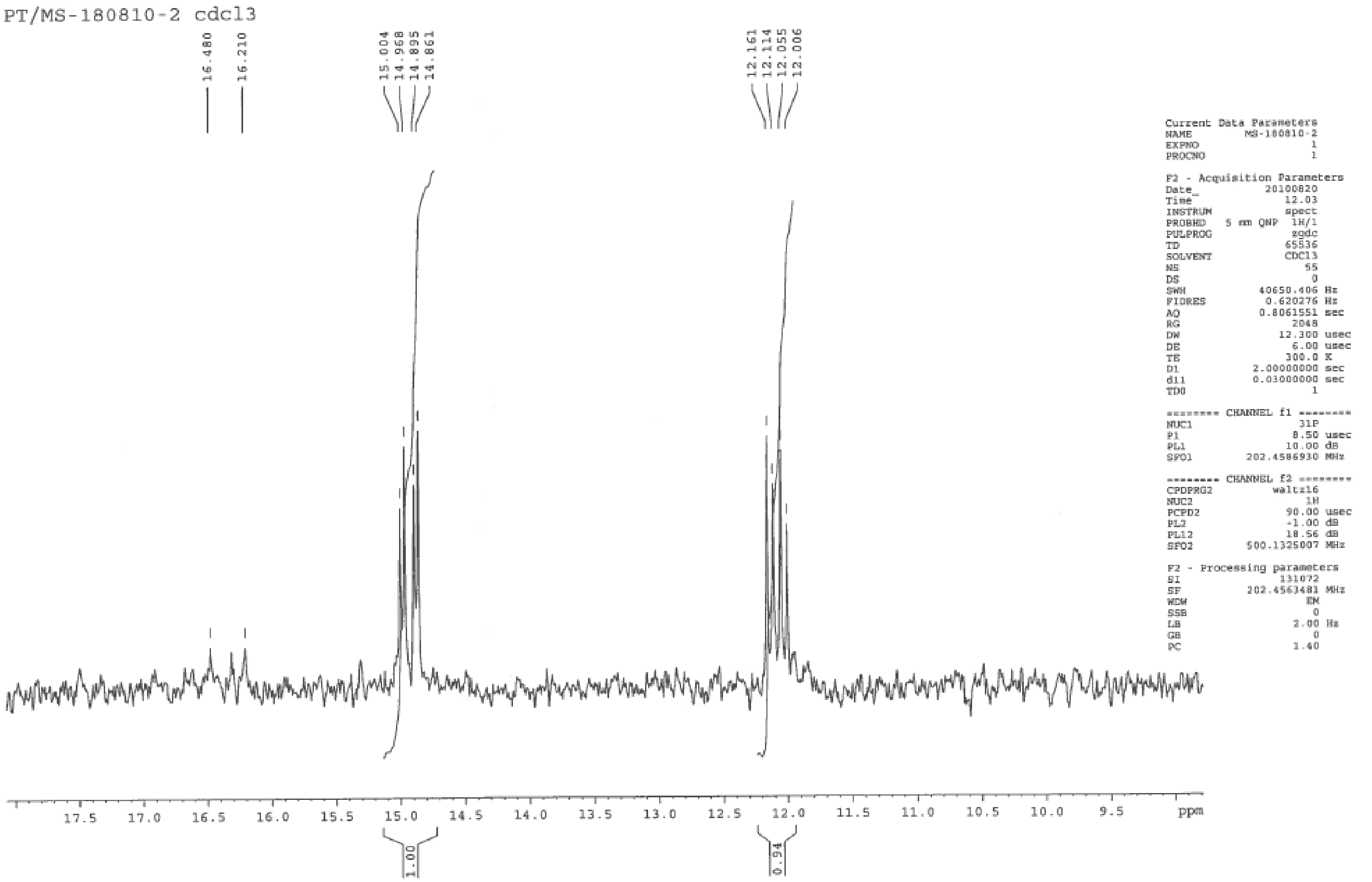
PT/MS-180810-2 cdcl3
16.480
16.210
15.004
14.968
14.895
14.861
12.161
12.114
12.055
12.006
Current Data Parameters
NAME MS-180810-2
EXPNO 1
PROCNO 1
F2 - Acquisition Parameters
Date_ 20100820
Time 12.03
INSTRUM spect
PROBHD 5 mm QNP 1H/1
PULPROG zgdc
TD 65536
SOLVENT CDCl3
NS 55
DS 0
SWH 40650.406 Hz
FIDRES 0.620276 Hz
AQ 0.8061551 sec
RG 2048
DW 12.300 usec
DE 6.00 usec
TE 300.0 K
D1 2.00000000 sec
d11 0.03000000 sec
TD0 1
======== CHANNEL f1 ========
NUC1 31P
P1 8.50 usec
PL1 10.00 dB
SFO1 202.4586930 MHz
======== CHANNEL f2 ========
CPDPRG2 waltz16
NUC2 1H
PCPD2 90.00 usec
PL2 -1.00 dB
PL12 18.56 dB
SFO2 500.1325007 MHz
F2 - Processing parameters
SI 131072
SF 202.4563481 MHz
WDW EM
SSB 0
LB 2.00 Hz
GB 0
PC 1.40
17.5
17.0
16.5
16.0
15.5
15.0
14.5
14.0
13.5
13.0
12.5
12.0
11.5
11.0
10.5
10.0
9.5
ppm
1.00
0.94

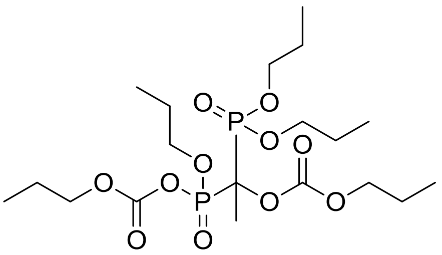

3c

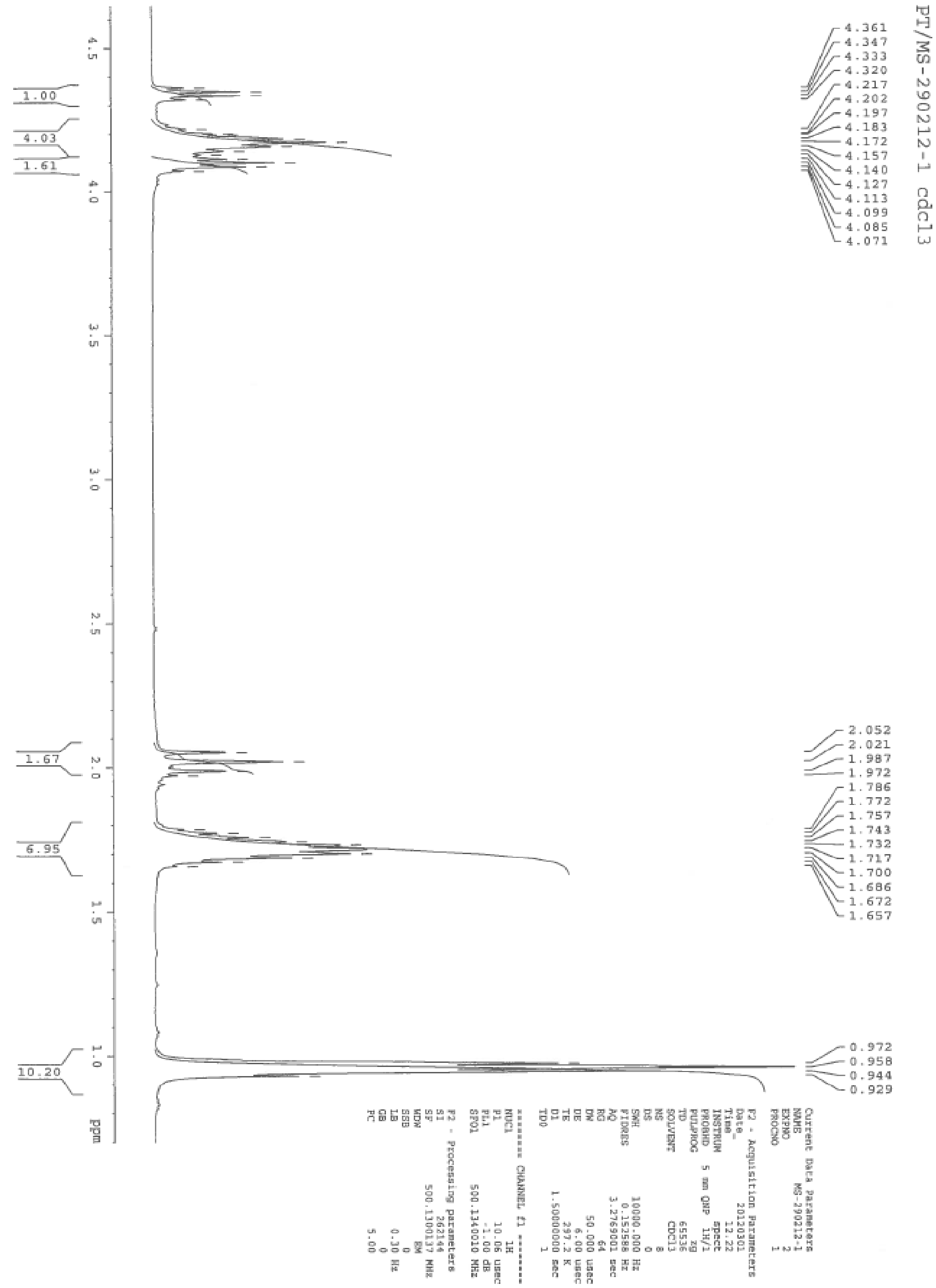

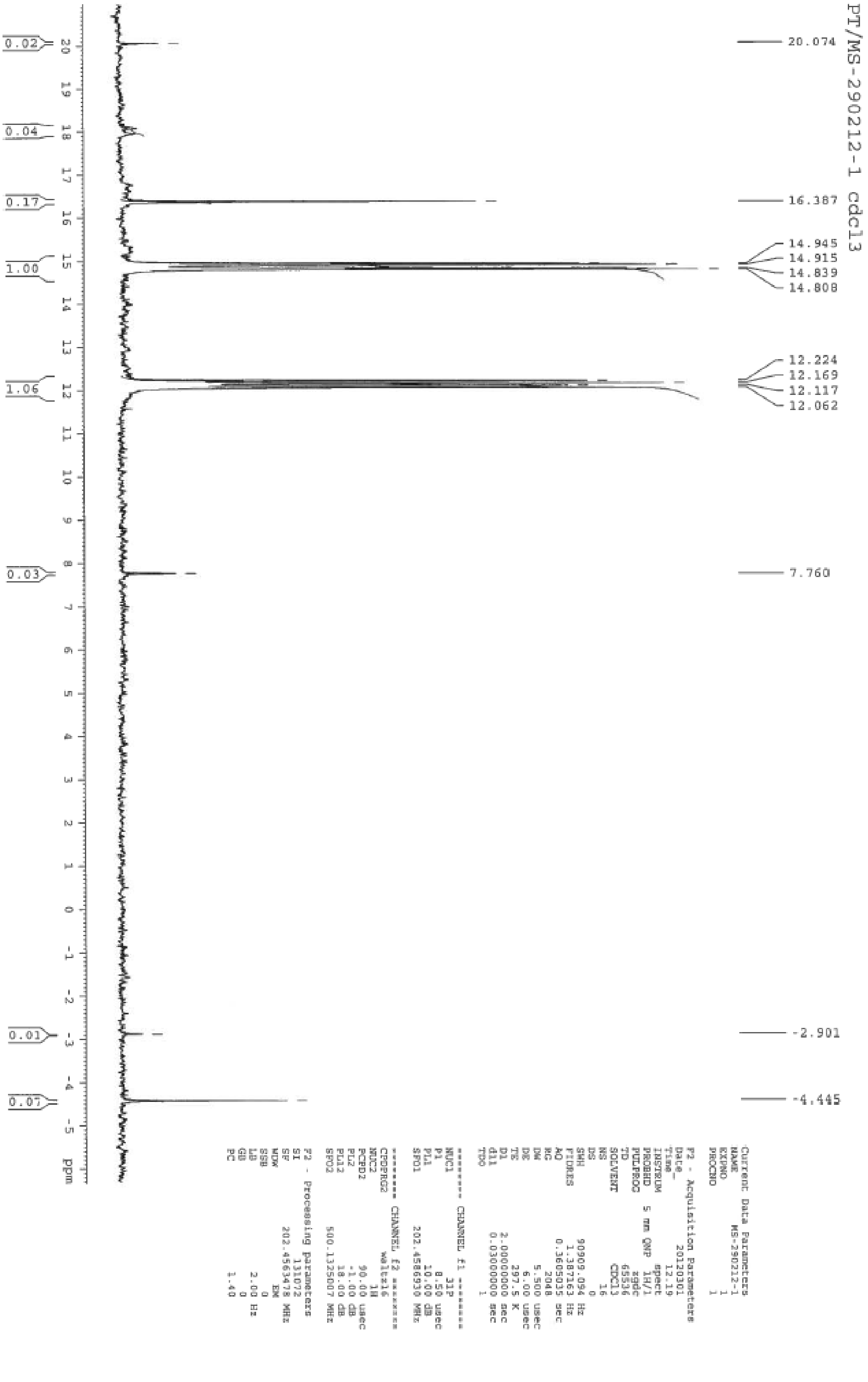
PT/MS-290212-1 cdcl3
20.074
16.387
14.945
14.915
14.839
14.808
12.224
12.169
12.117
12.062
7.760
-2.901
-4.445
Current Data Parameters
NAME MS-290212-1
EXPNO 1
PROCNO 1
F2 - Acquisition Parameters
Date_ 20120301
Time 12.19
INSTRUM spect
PROBHD 5 mm QNP 1H/1
PULPROG zgdc
TD 65536
SOLVENT CDCl3
NS 16
DS 0
SWH 90909.094 Hz
FIDRES 1.387163 Hz
AQ 0.3605035 sec
RG 2048
DW 5.500 usec
DE 6.00 usec
TE 297.5 K
D1 2.00000000 sec
d11 0.03000000 sec
TD0 1
======== CHANNEL f1 ========
NUC1 31P
P1 8.50 usec
PL1 10.00 dB
SFO1 202.4586930 MHz
======== CHANNEL f2 ========
CPDPRG2 waltz16
NUC2 1H
PCPD2 90.00 usec
PL2 -1.00 dB
PL12 18.00 dB
SFO2 500.1325007 MHz
F2 - Processing parameters
SI 131072
SF 202.4563478 MHz
WDW EM
SSB 0
LB 2.00 Hz
GB 0
PC 1.40
20 19 18 17 16 15 14 13 12 11 10 9 8 7 6 5 4 3 2 1 0 -1 -2 -3 -4 -5 ppm
0.02
0.04
0.17
1.00
1.06
0.03
0.01
0.07

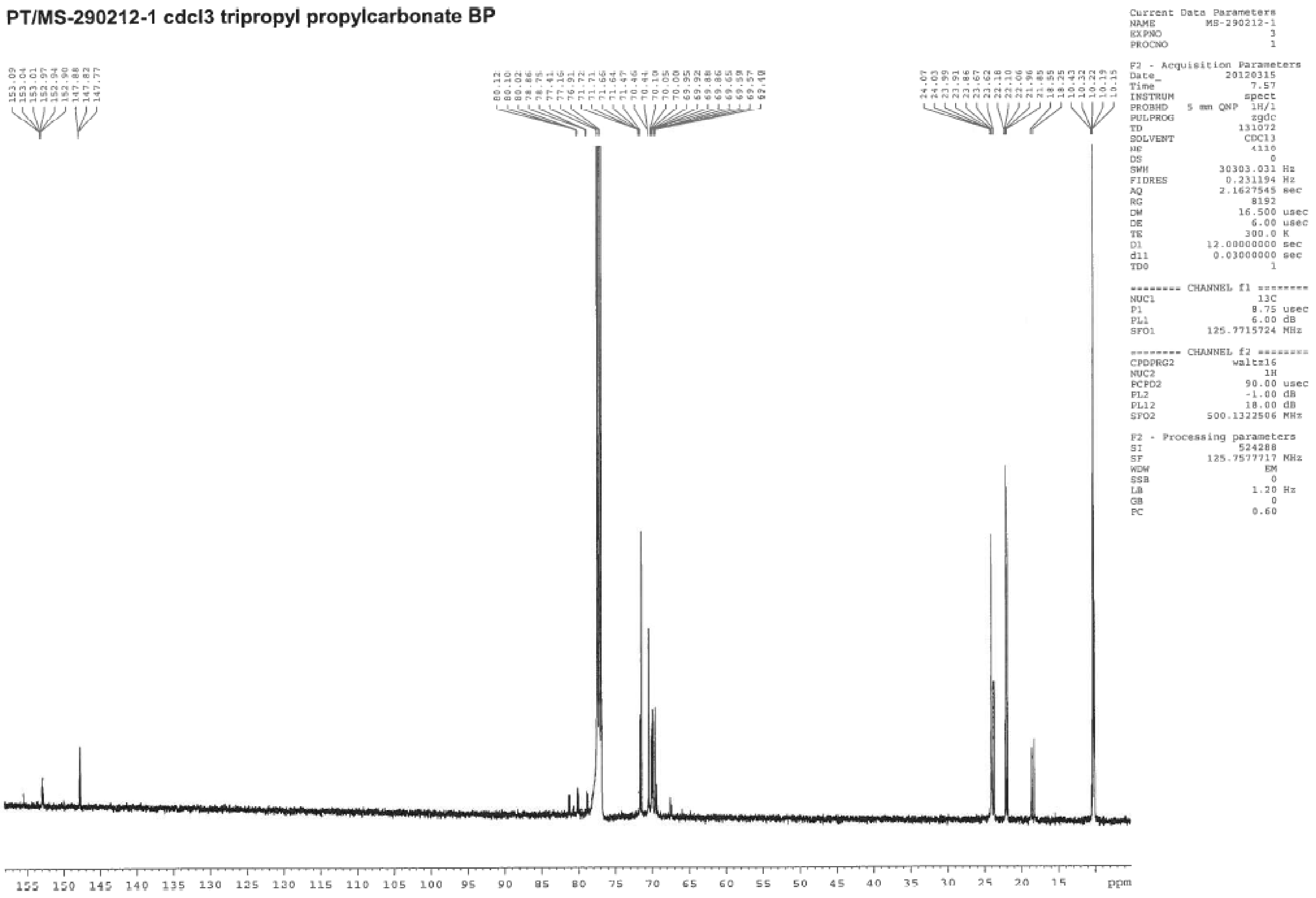
PT/MS-290212-1 cdcl3 tripropyl propylcarbonate BP
153.09
153.04
153.01
152.97
152.94
152.90
147.88
147.82
147.77
80.12
80.10
80.02
78.86
78.75
77.41
77.16
76.91
71.72
71.71
71.66
71.64
71.47
70.46
70.44
70.10
70.05
70.00
69.95
69.92
69.88
69.86
69.65
69.59
69.57
67.40
24.07
24.03
23.99
23.91
23.86
23.67
23.62
22.18
22.10
22.06
21.96
21.85
18.55
18.25
10.43
10.32
10.22
10.19
10.15
Current Data Parameters
NAME MS-290212-1
EXPNO 3
PROCNO 1
F2 - Acquisition Parameters
Date_ 20120315
Time 7.57
INSTRUM spect
PROBHD 5 mm QNP 1H/1
PULPROG zgdc
TD 131072
SOLVENT CDCl3
NS 4110
DS 0
SWH 30303.031 Hz
FIDRES 0.231194 Hz
AQ 2.1627545 sec
RG 8192
DW 16.500 usec
DE 6.00 usec
TE 300.0 K
D1 12.00000000 sec
d11 0.03000000 sec
TD0 1
======== CHANNEL f1 ========
NUC1 13C
P1 8.75 usec
PL1 6.00 dB
SFO1 125.7715724 MHz
======== CHANNEL f2 ========
CPDPRG2 waltz16
NUC2 1H
PCPD2 90.00 usec
PL2 -1.00 dB
PL12 18.00 dB
SFO2 500.1322506 MHz
F2 - Processing parameters
SI 524288
SF 125.7577717 MHz
WDW EM
SSB 0
LB 1.20 Hz
GB 0
PC 0.60
155 150 145 140 135 130 125 120 115 110 105 100 95 90 85 80 75 70 65 60 55 50 45 40 35 30 25 20 15 ppm

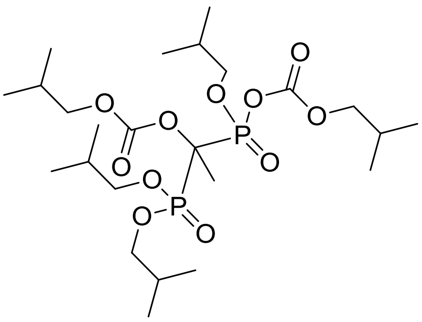

3d

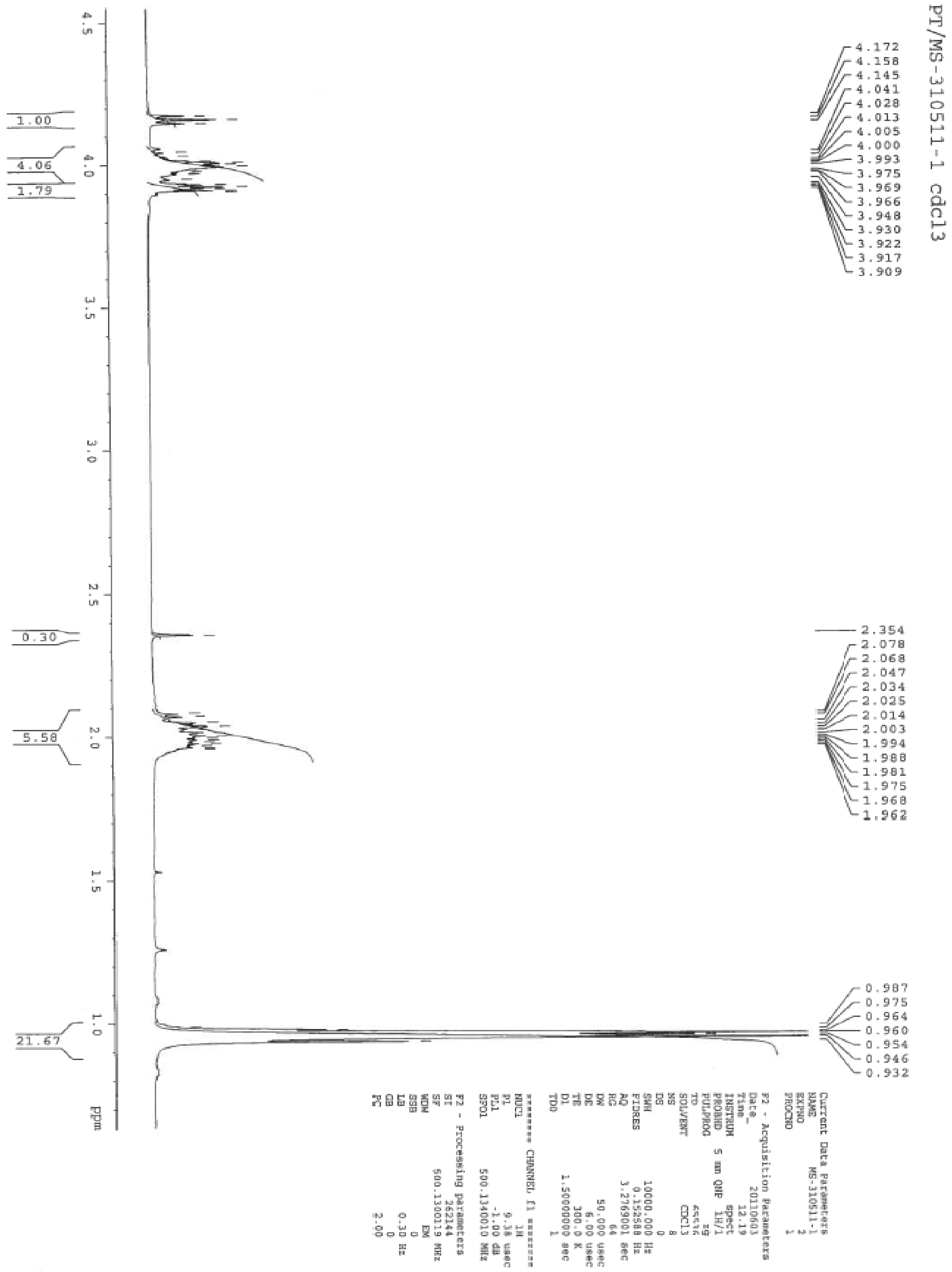
PT/MS-310511-1 cdcl3
4.172
4.158
4.145
4.041
4.028
4.013
4.005
4.000
3.993
3.975
3.969
3.966
3.948
3.930
3.922
3.917
3.909
2.354
2.078
2.068
2.047
2.034
2.025
2.014
2.003
1.994
1.988
1.981
1.975
1.968
1.962
0.987
0.975
0.964
0.960
0.954
0.946
0.932
1.00
4.06
1.79
0.30
5.58
21.67
4.5
4.0
3.5
3.0
2.5
2.0
1.5
1.0
ppm

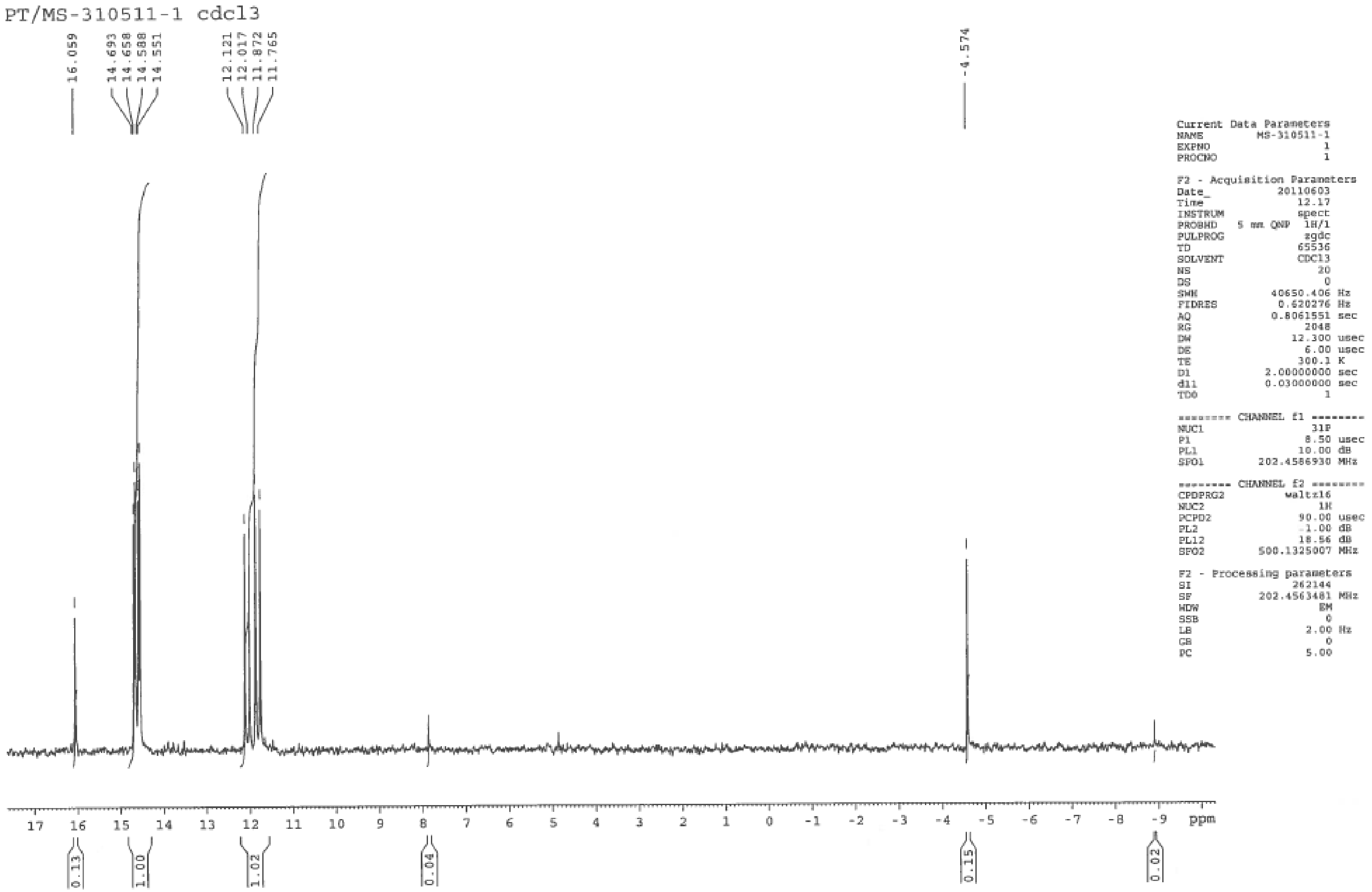
PT/MS-310511-1 cdcl3
16.059
14.693
14.658
14.588
14.551
12.121
12.017
11.872
11.765
-4.574
0.13
1.00
1.02
0.04
0.15
0.02
17 16 15 14 13 12 11 10 9 8 7 6 5 4 3 2 1 0 -1 -2 -3 -4 -5 -6 -7 -8 -9 ppm
Current Data Parameters
NAME MS-310511-1
EXPNO 1
PROCNO 1
F2 - Acquisition Parameters
Date_ 20110603
Time 12.17
INSTRUM spect
PROBHD 5 mm QNP 1H/1
PULPROG zgdc
TD 65536
SOLVENT CDCl3
NS 20
DS 0
SWH 40650.406 Hz
FIDRES 0.620276 Hz
AQ 0.8061551 sec
RG 2048
DW 12.300 usec
DE 6.00 usec
TE 300.1 K
D1 2.00000000 sec
d11 0.03000000 sec
TD0 1
======== CHANNEL f1 ========
NUC1 31P
P1 8.50 usec
PL1 10.00 dB
SFO1 202.4586930 MHz
======== CHANNEL f2 ========
CPDPRG2 waltz16
NUC2 1H
PCPD2 90.00 usec
PL2 -1.00 dB
PL12 18.56 dB
SFO2 500.1325007 MHz
F2 - Processing parameters
SI 262144
SF 202.4563481 MHz
WDW EM
SSB 0
LB 2.00 Hz
GB 0
PC 5.00

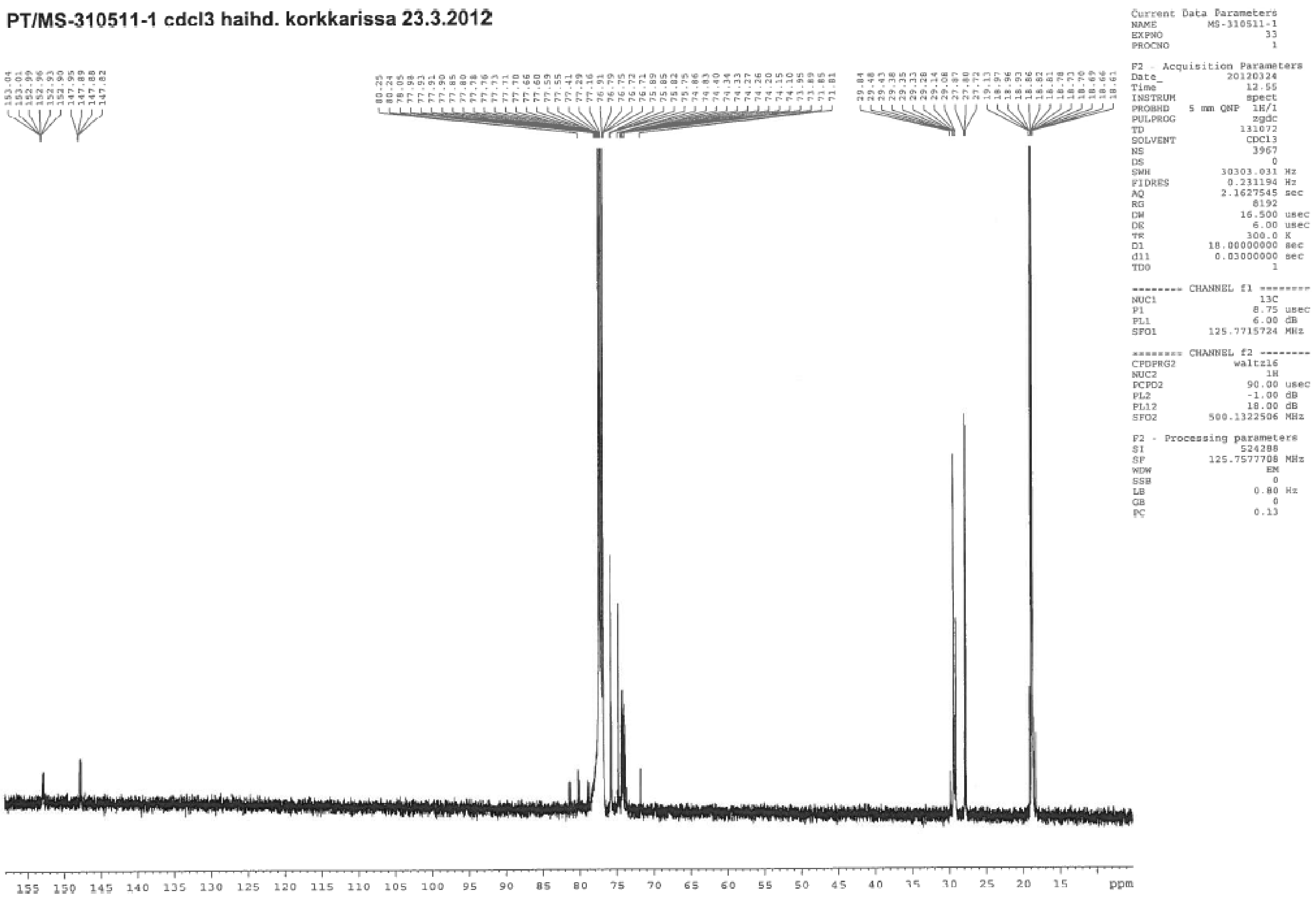
PT/MS-310511-1 cdcl3 haihd. korkkarissa 23.3.2012
153.04
153.01
152.99
152.96
152.93
152.90
147.95
147.89
147.88
147.82
80.25
80.24
78.05
77.98
77.93
77.91
77.90
77.85
77.80
77.78
77.76
77.73
77.71
77.70
77.66
77.60
77.59
77.55
77.41
77.29
77.16
76.91
76.79
76.75
76.72
76.71
75.89
75.85
75.82
75.75
74.86
74.83
74.40
74.34
74.33
74.27
74.26
74.20
74.15
74.10
73.95
73.89
71.85
71.81
29.84
29.48
29.43
29.38
29.35
29.33
29.28
29.14
29.08
27.87
27.80
27.72
19.13
18.97
18.96
18.93
18.86
18.82
18.81
18.78
18.73
18.70
18.69
18.66
18.61
155 150 145 140 135 130 125 120 115 110 105 100 95 90 85 80 75 70 65 60 55 50 45 40 35 30 25 20 15 ppm
Current Data Parameters
NAME MS-310511-1
EXPNO 33
PROCNO 1
F2 - Acquisition Parameters
Date_ 20120324
Time 12.55
INSTRUM spect
PROBHD 5 mm QNP 1H/1
PULPROG zgdc
TD 131072
SOLVENT CDCl3
NS 3967
DS 0
SWH 30303.031 Hz
FIDRES 0.231194 Hz
AQ 2.1627545 sec
RG 8192
DW 16.500 usec
DE 6.00 usec
TE 300.0 K
D1 18.00000000 sec
d11 0.03000000 sec
TD0 1
======== CHANNEL f1 ========
NUC1 13C
P1 8.75 usec
PL1 6.00 dB
SFO1 125.7715724 MHz
======== CHANNEL f2 ========
CPDPRG2 waltz16
NUC2 1H
PCPD2 90.00 usec
PL2 -1.00 dB
PL12 18.00 dB
SFO2 500.1322506 MHz
F2 - Processing parameters
SI 524288
SF 125.7577708 MHz
WDW EM
SSB 0
LB 0.80 Hz
GB 0
PC 0.13

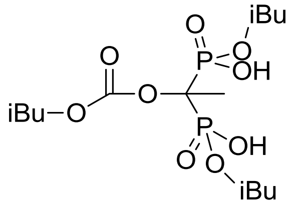

2

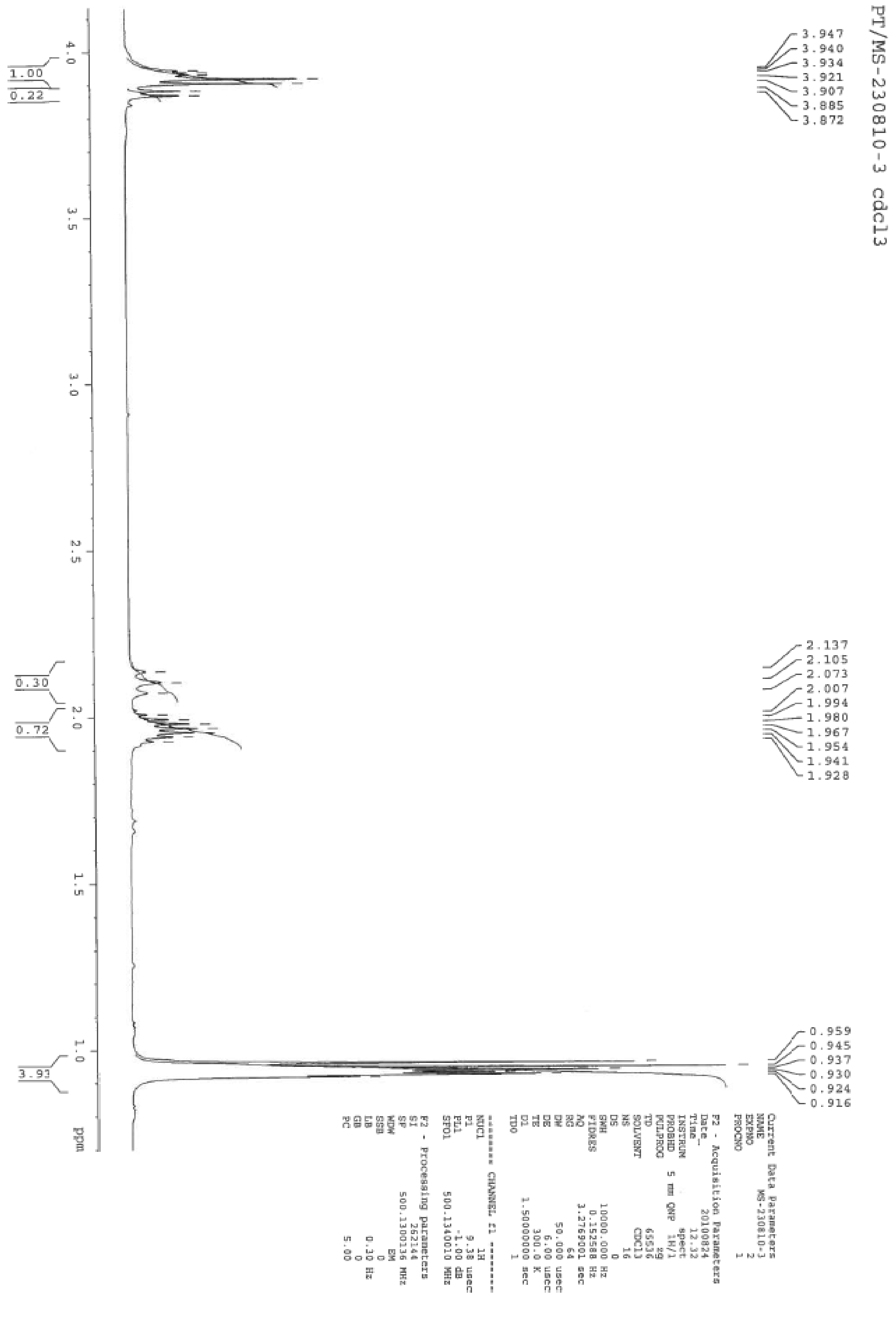

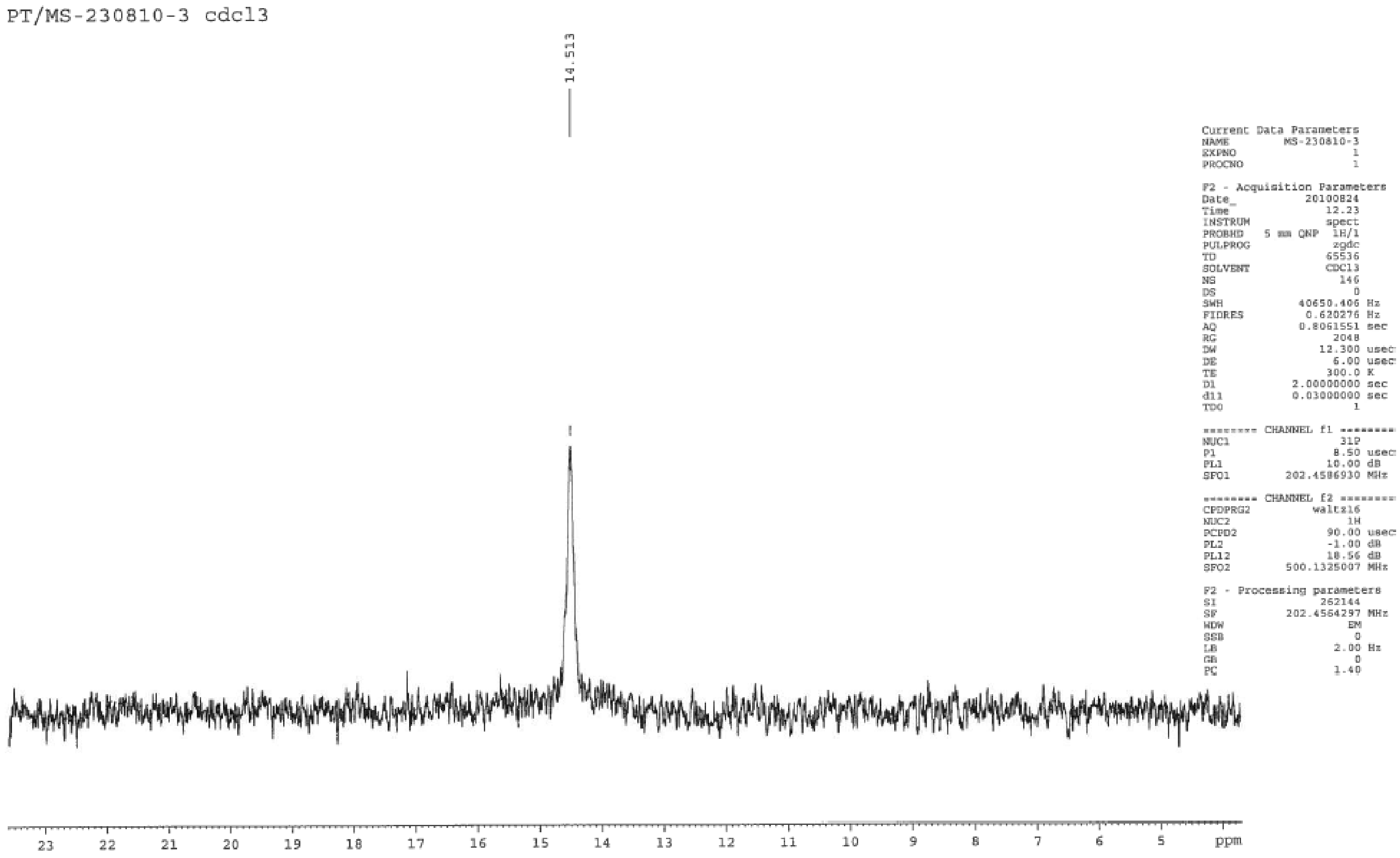
PT/MS-230810-3 cdcl3
14.513
Current Data Parameters
NAME MS-230810-3
EXPNO 1
PROCNO 1
F2 - Acquisition Parameters
Date_ 20100824
Time 12.23
INSTRUM spect
PROBHD 5 mm QNP 1H/1
PULPROG zgdc
TD 65536
SOLVENT CDCl3
NS 146
DS 0
SWH 40650.406 Hz
FIDRES 0.620276 Hz
AQ 0.8061551 sec
RG 2048
DW 12.300 usec
DE 6.00 usec
TE 300.0 K
D1 2.00000000 sec
d11 0.03000000 sec
TD0 1
======== CHANNEL f1 ========
NUC1 31P
P1 8.50 usec
PL1 10.00 dB
SFO1 202.4586930 MHz
======== CHANNEL f2 ========
CPDPRG2 waltz16
NUC2 1H
PCPD2 90.00 usec
PL2 -1.00 dB
PL12 18.56 dB
SFO2 500.1325007 MHz
F2 - Processing parameters
SI 262144
SF 202.4564297 MHz
WDW EM
SSB 0
LB 2.00 Hz
GB 0
PC 1.40
23 22 21 20 19 18 17 16 15 14 13 12 11 10 9 8 7 6 5 ppm

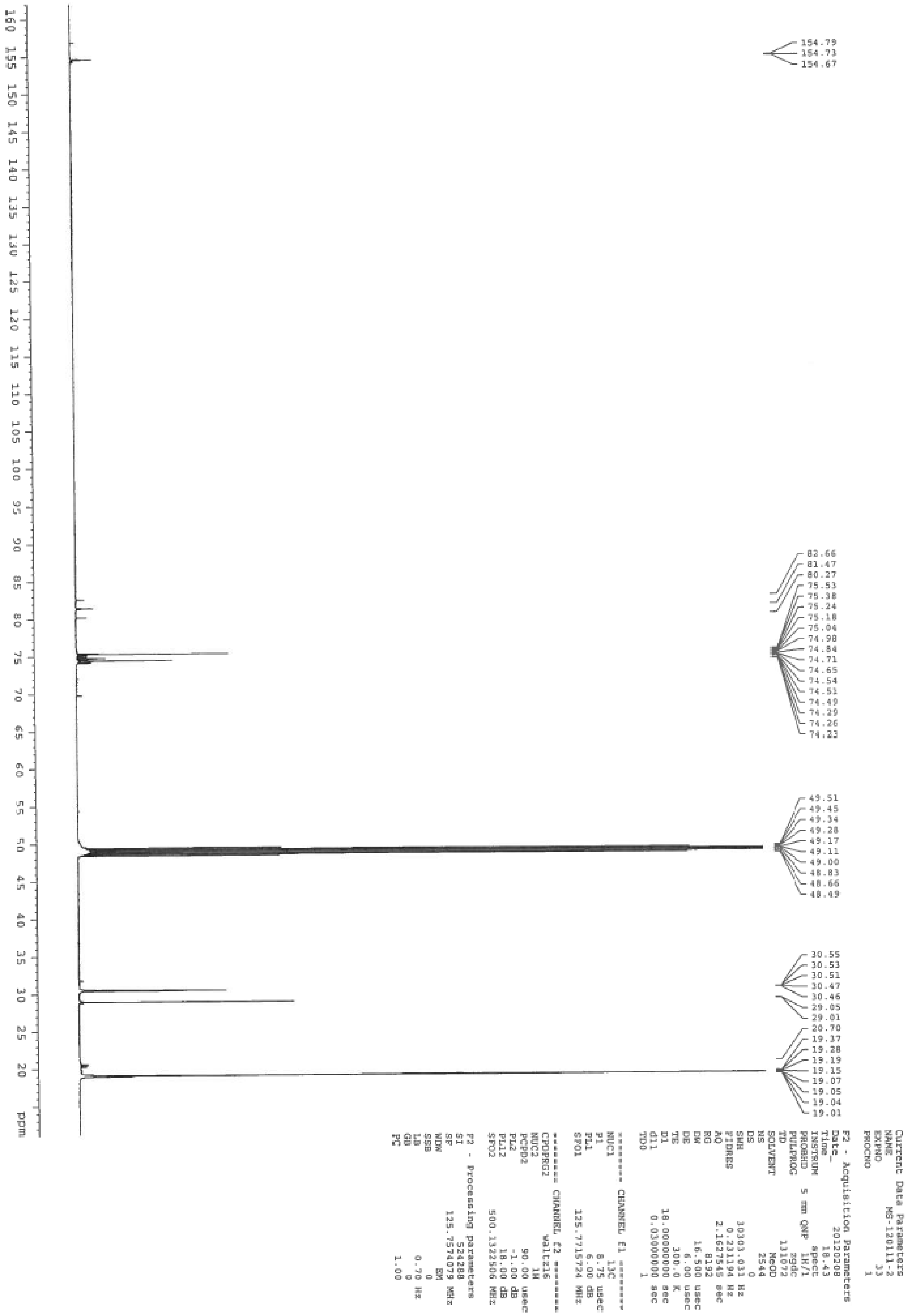
154.79
154.73
154.67
82.66
81.47
80.27
75.53
75.38
75.24
75.18
75.04
74.98
74.84
74.71
74.65
74.54
74.51
74.49
74.29
74.26
74.23
49.51
49.45
49.34
49.28
49.17
49.11
49.00
48.83
48.66
48.49
30.55
30.53
30.51
30.47
30.46
29.05
29.01
20.70
19.37
19.28
19.19
19.15
19.07
19.05
19.04
19.01
Current Data Parameters
NAME MS-120111-2
EXPNO 33
PROCNO 1
F2 - Acquisition Parameters
Date_ 20120208
Time 18.43
INSTRUM spect
PROBHD 5 mm QNP 1H/1
PULPROG zgdc
TD 131072
SOLVENT MeOD
NS 2544
DS 0
SWH 30303.031 Hz
FIDRES 0.231194 Hz
AQ 2.1627545 sec
RG 8192
DW 16.500 usec
DE 6.00 usec
TE 300.0 K
D1 18.00000000 sec
d11 0.03000000 sec
TD0 1
======== CHANNEL f1 ========
NUC1 13C
P1 8.75 usec
PL1 6.00 dB
SFO1 125.7715724 MHz
======== CHANNEL f2 ========
CPDPRG2 waltz16
NUC2 1H
PCPD2 90.00 usec
PL2 -1.00 dB
PL12 18.00 dB
SFO2 500.1322506 MHz
F2 - Processing parameters
SI 524288
SF 125.7574079 MHz
WDW EM
SSB 0
LB 0.70 Hz
GB 0
PC 1.00
160 155 150 145 140 135 130 125 120 115 110 105 100 95 90 85 80 75 70 65 60 55 50 45 40 35 30 25 20 ppm

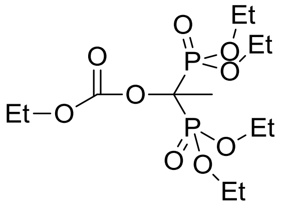
O
Et
Et
O
P
O
Et—O
O
P
O
Et
O
O
Et

4

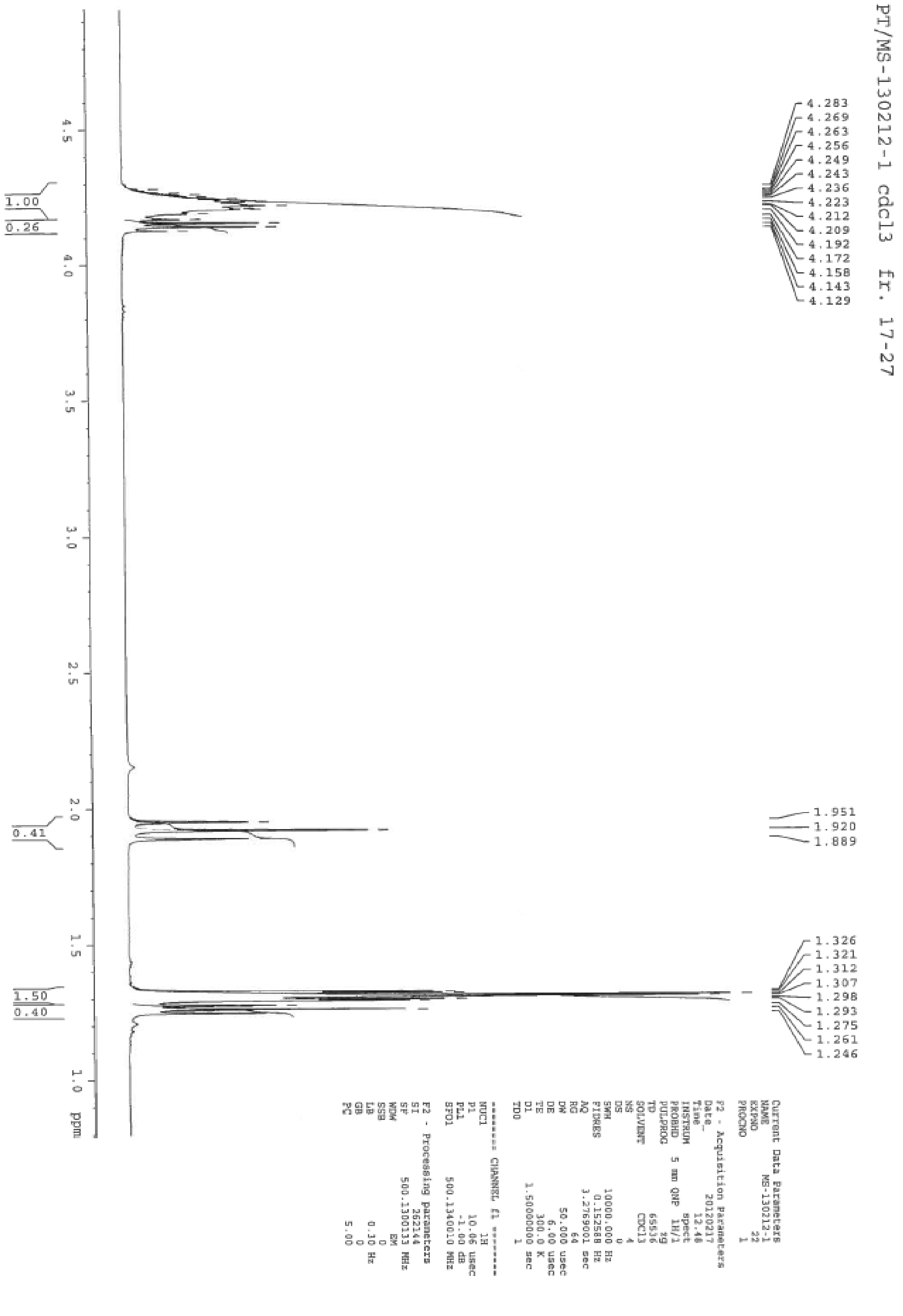
PT/MS-130212-1 cdcl3 fr. 17-27
4.283
4.269
4.263
4.256
4.249
4.243
4.236
4.223
4.212
4.209
4.192
4.172
4.158
4.143
4.129
1.951
1.920
1.889
1.326
1.321
1.312
1.307
1.298
1.293
1.275
1.261
1.246
1.00
0.26
0.41
1.50
0.40
4.5
4.0
3.5
3.0
2.5
2.0
1.5
1.0
ppm

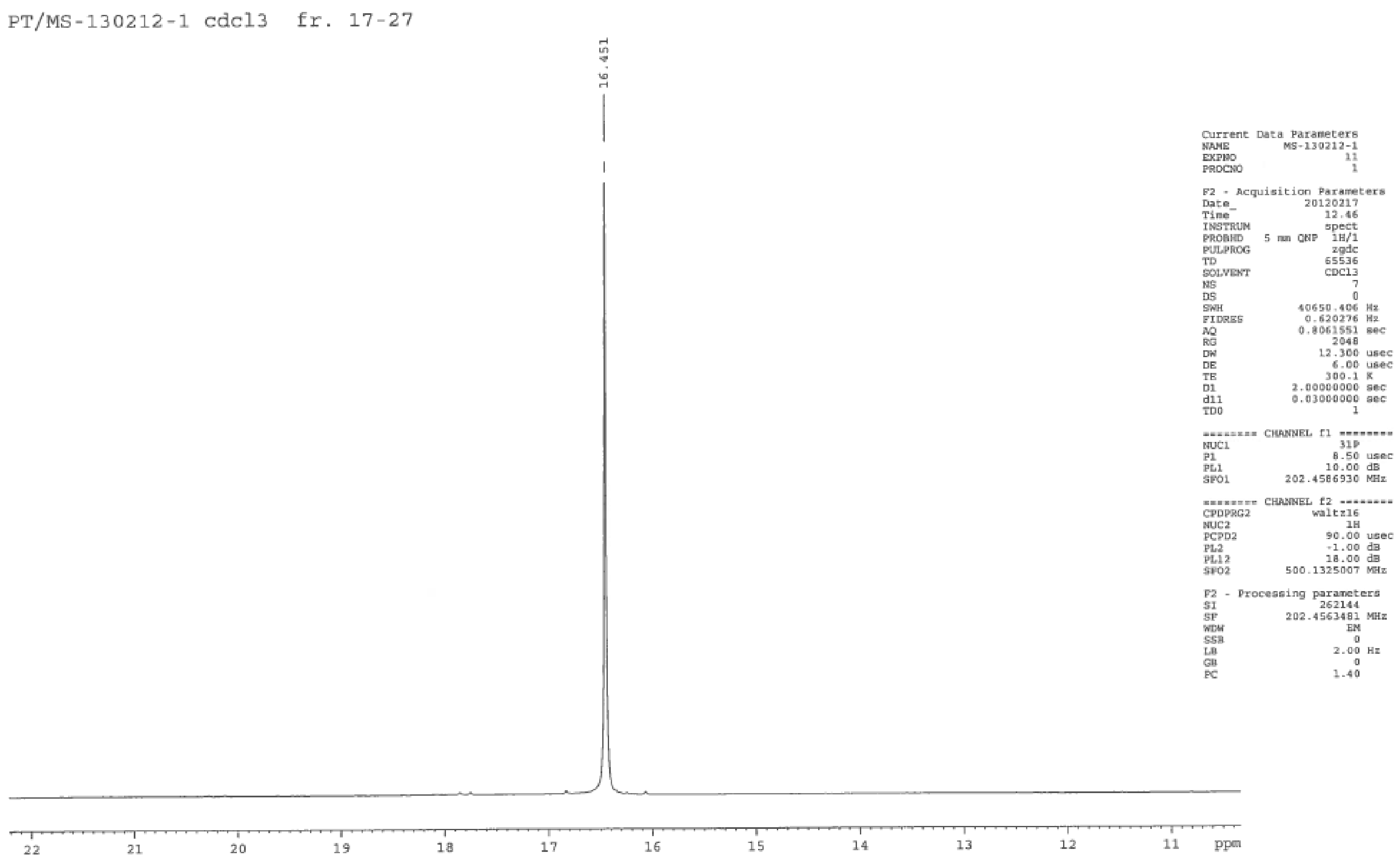
PT/MS-130212-1 cdcl3 fr. 17-27
16.451
22 21 20 19 18 17 16 15 14 13 12 11 ppm
Current Data Parameters
NAME MS-130212-1
EXPNO 11
PROCNO 1
F2 - Acquisition Parameters
Date_ 20120217
Time 12.46
INSTRUM spect
PROBHD 5 mm QNP 1H/1
PULPROG zgdc
TD 65536
SOLVENT CDCl3
NS 7
DS 0
SWH 40650.406 Hz
FIDRES 0.620276 Hz
AQ 0.8061551 sec
RG 2048
DW 12.300 usec
DE 6.00 usec
TE 300.1 K
D1 2.00000000 sec
d11 0.03000000 sec
TD0 1
======== CHANNEL f1 ========
NUC1 31P
P1 8.50 usec
PL1 10.00 dB
SFO1 202.4586930 MHz
======== CHANNEL f2 ========
CPDPRG2 waltz16
NUC2 1H
PCPD2 90.00 usec
PL2 -1.00 dB
PL12 18.00 dB
SFO2 500.1325007 MHz
F2 - Processing parameters
SI 262144
SF 202.4563481 MHz
WDW EM
SSB 0
LB 2.00 Hz
GB 0
PC 1.40

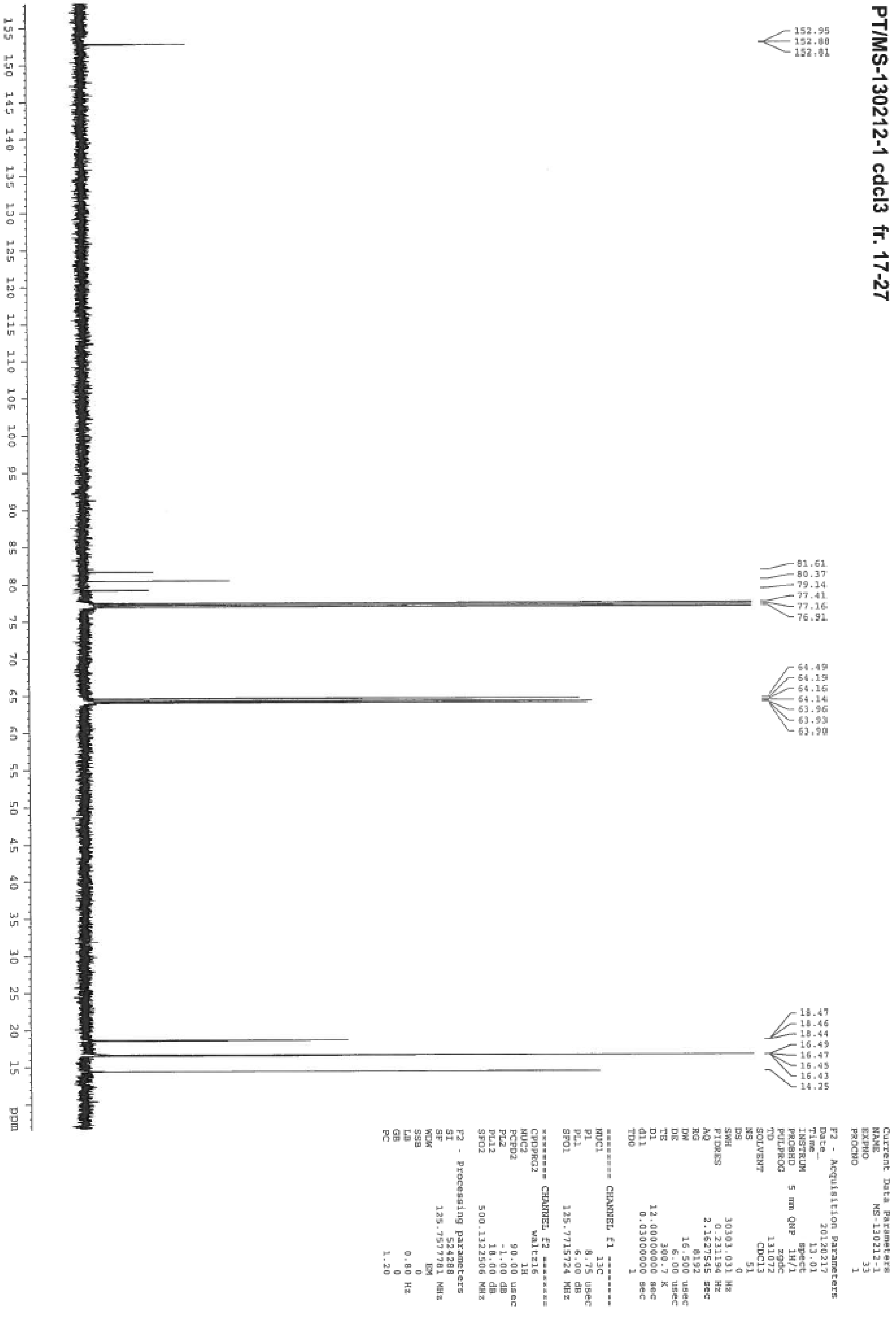
PT/MS-130212-1 cdcl3 fr. 17-27
152.95
152.88
152.81
81.61
80.37
79.14
77.41
77.16
76.91
64.49
64.19
64.16
64.14
63.96
63.93
63.90
18.47
18.46
18.44
16.49
16.47
16.45
16.43
14.35
ppm
